# Fieldable isothermal nucleic acid test for rapid semi-quantitative visual readout of enterococci in recreational waters

**DOI:** 10.7717/peerj.21310

**Published:** 2026-05-22

**Authors:** Anirudh Sudarshan, Meredith G. Rillera, Nicholas Tran, Timothy E. Riedel, Andrew D. Ellington, Sanchita Bhadra

**Affiliations:** 1 Department of Molecular Biosciences, College of Natural Sciences, University of Texas at Austin, Austin, TX, United States of America; 2 College of Natural Sciences, University of Texas at Austin, Austin, TX, United States of America

**Keywords:** Loop-mediated isothermal amplification, Oligonucleotide strand displacement probes, Amplicon competition, Fecal indicator bacteria, Water quality monitoring, Low resource nucleic acid test, Thresholded LAMP-OSD, Nucleic acid semi-quantitation *via* single visual readout, Enterococci contamination monitoring

## Abstract

Enterococci are fecal indicator bacteria whose presence in water suggests the potential for gastrointestinal pathogens. Culture-based detection usually needs 18-48 hours and may require expensive apparatus and proprietary media. Molecular tests based on qPCR can be technically difficult and require complex devices. Loop-mediated isothermal amplification (LAMP) tests need minimal instrumentation, but they only qualitatively indicate the presence or absence of enterococci, making comparison with quantitative contamination standards difficult. Here, we demonstrate semi-quantitative ‘thresholded’ LAMP via competition with pre-defined false targets to estimate the extent of enterococci contamination in water. The assay needs only one hour of incubation at a single temperature following which a simple visual examination of an oligonucleotide strand displacement (OSD) probe’s fluorescence allows order of magnitude scale estimation of enterococci contamination, correlating closely with the outcomes of both qPCR and plate cultures. Environmental water samples with low (undetectable by qPCR), medium (10s of DNA copies/mL), and high (1,000s of DNA copies/mL) enterococci contamination could be readily distinguished by thresholded LAMP-OSD, without interference from non-specific signals. The simplicity of implementing thresholded LAMP-OSD makes it well suited for point-of-need and low-cost water quality monitoring.

## Introduction

Exposure to gastrointestinal pathogens in water can lead to significant illness and financial burden (De Florio-Barker et al., 2018). Traditionally water quality assessment and standards for recreational waters have been based on fecal indicator bacteria (FIB) like the fecal coliform, *Escherichia coli*, and enterococci, due to their prevalence in human and animal feces (Holcomb & Stewart, 2020). Current methods for quantitating FIB typically involve culture-based techniques, such as membrane filtration (MF) and most probable number (MPN) testing (McConn et al., 2024). Culture-based methods, though theoretically straightforward and cost-effective, require 18–48 h to yield results and are prone to contamination and both false positive and false negative errors (McConn et al., 2024). Moreover, in practice, both MF and MPN require a high level of expertise for sample preparation and dilution and interpretation of results (Sikder et al., 2021). Similarly, expensive specialized or proprietary enrichment media and automated systems, such as the IDEXX Quanti-Tray, may be required for culture-based sample analysis and present a significant cost barrier. Overall, these limitations hamper the use of MPN/MF for field research and testing. Portions of this text were previously published as part of a preprint (https://doi.org/10.1101/2025.04.20.649722). 

Recently polymerase chain reaction (PCR) and quantitative polymerase chain reaction (qPCR)-based tests have been developed that allow for microbial source tracking (MST) between animals (Boehm et al., 2013), and these could obviously be used for monitoring water quality. However, PCR-based tests require DNA extraction, have high reagents costs (Riedel et al., 2014), demand expert labor to set up and analyze, and need complex instrumentation (Moehling et al., 2021), making regular monitoring not only expensive but slow. The typical documented sample to answer time with the USEPA enterococci qPCR method is 3.5 to 4 h after receipt of sample with some protocol alterations reducing testing time by 20 min (Saleem et al., 2023; Sivaganesan et al., 2025). While this timeframe can allow same-day actionable water quality monitoring, it is only practical if the water body is within ∼1 h transport distance from the testing lab and samples can be collected by 7:00 a.m (Saleem et al., 2023). Consequently, <1% of beach water microbial testing in the United States is performed using qPCR (Shrestha & Dorevitch, 2020).

Loop-mediated isothermal amplification (LAMP) of nucleic acids offers a promising, fieldable alternative to PCR (Notomi et al., 2000; Jiang et al., 2018). LAMP can isothermally produce 10^9^ to 10^10^ copies of target nucleic acids, usually within one hour (Notomi et al., 2000), and can be used to directly analyze crudely processed samples without laborious nucleic acid purification or instrumentation (Jiang et al., 2018; Bhadra, Esteve-Gasent & Ellington, 2023; Bhadra et al., 2018a; Bhadra et al., 2018b). However, since LAMP provides a qualitative (yes/no) readout of a nucleic acid sequence this can be limiting for water monitoring, where quality assessments are based on measuring elevations in FIB levels.

By developing oligonucleotide strand displacement (OSD) probes we further improved the utility of LAMP by decreasing false positives from spurious amplicons (Bhadra et al., 2021b; Jiang et al., 2015). The simplest OSD comprises a fluorophore-labeled long strand bound to a quencher-labeled short strand whose displacement by a complementary LAMP amplicon results in fluorescence (Jiang et al., 2015). OSDs have been previously used to identify amplicons and single nucleotide polymorphisms (SNPs) (Jiang et al., 2015; Bhadra et al., 2015), compute multiplex amplicons into Boolean output (Bhadra, Esteve-Gasent & Ellington, 2023; Bhadra et al., 2018b; Bhadra et al., 2021b), transduce LAMP to color or glucose (Du et al., 2015), and most importantly for this study semi-quantify target templates from a single endpoint readout by thresholding OSD signal by using competing OSD-unresponsive false templates (Jiang et al., 2017).

LAMP-OSD can be used for the direct analysis of heat-lysed *E. coli* as well as the rapid semi-quantitation of *Bacteroides* HF183 sequences of human fecal origin in sewage-contaminated water (Jiang et al., 2018). We have now also re-engineered the enterococci LAMP assay for template semi-quantitation on an order of magnitude scale with the added simplicity of a single endpoint visual readout, thereby minimizing instrumentation and facilitating portability for analysis of environmental water samples.

## Materials & Methods

### Chemicals and reagents

All chemicals were purchased from Sigma-Aldrich (St. Louis, MO, USA) unless indicated otherwise. All enzymes were purchased from New England Biolabs (NEB, Ipswich, MA, USA). All oligonucleotides and synthetic DNA were purchased from Integrated DNA Technologies (IDT, Coralville, IA, USA) or Twist Biosciences (Quincy, MA, USA) (Table S1). *Enterococcus faecalis* NCTC 775 strain was purchased from ATCC (Manassas, Virginia, USA) and *E. faecalis* culti loops (Culti-Loops *Enterococcus faecalis* ATCC 19433) were obtained from Thermo Fisher Scientific (Waltham, MA, USA).

### *Enterococcus faecalis* culture

*E. faecalis* was grown overnight at 37 °C using Brain Heart Infusion Agar or Broth (Hardy Diagnostics, Santa Maria, CA, USA). Liquid cultures were aerated at 250 rpm. Prior to use in LAMP-OSD analysis, 1:300 sub-cultures of the overnight cultures were grown in fresh culture medium for four hours in a 37 °C shaker.

### OSD probe design

Using our previously described design rules (Jiang et al., 2015), the NUPACK software (Pasadena, CA, USA) (Zadeh et al., 2011), and the Benchling platform (San Francisco, CA, USA), an OSD probe was designed for a previously reported *E. faecalis* 23S rRNA gene-specific LAMP assay (Martzy et al., 2017). The hemiduplex OSD consisted of a 40-nt long 5′-end fluorescein-labeled long strand, designed to hybridize to a 37-nt long region in the loop sequence between the B1 and B2c regions of the LAMP amplicon (Fig. S1). The 3′-end OH group of this strand was blocked against extension by appending an inverted dT residue. A 25-nt long portion of the OSD binding region of the LAMP amplicon was also recognized by the Loop B primer. The OSD short strand modified with a 3′-end Iowa Black FQ quencher was designed for complementarity to 29-nt at the 5′-end of the OSD long strand. The first three base pairs of this hemiduplex, abutting the fluorophore:quencher pair, served as a clamp and were unrelated to the *Enterococcus* sequence. This ensures greater thermodynamic stability of the OSD probe *versus* pairing of the OSD short strand with the 25-nt long Loop B primers. The hemiduplex OSD was pre-annealed by mixing 1 µM of the long strand with 5 µM of the short strand in 1X isothermal buffer (NEB: 20 mM Tris–HCl, 10 mM (NH_4_)_2_SO_4_, 50 mM KCl, 2 mM MgSO_4_, 0.1% Tween^®^20, pH 8.8@25 °C) and incubating at 95 °C for 5 min followed by slow cooling (0.1 °C/sec) to room temperature.

### LAMP-OSD assay

LAMP-OSD reactions were set up based on our previously used visually-read LAMP-OSD conditions in 25 µL volume comprised of 1X isothermal buffer supplemented with 1.2 mM deoxyribonucleotides, 0.6 M betaine, 2 mM additional MgSO_4_, 2.4 µM each of FIP (forward inner primer) and BIP (backward inner primer), 1.2 µM each of Loop F and Loop B, and 0.6 µM each of F3 and B3 primers. All assays also received 16 units of Bst 2.0 DNA polymerase (NEB) along with 0.2 µM of OSD long strand pre-annealed with a 5-fold excess of the OSD short strand. In some LAMP assays, amplicon accumulation was evaluated by real-time measurement of dye intercalation by replacing OSD reporters in the reactions with 1X EvaGreen (Biotium, Fremont, CA, USA). Thresholded LAMP-OSD reactions also received indicated amounts of ‘false targets’ ranging from zero to the 10^10^ order of magnitude copies per reaction. The false targets were designed as synthetic *E. faecalis* 23S rDNA in which the OSD binding region was scrambled to prevent hybridization of OSD probes (and Loop B primers). The false targets can be amplified by the remaining five *E. faecalis* LAMP primers without generating corresponding OSD signals.

Performances of LAMP-OSD and thresholded LAMP-OSD assays were tested by adding indicated types and amounts of templates per reaction in 2.5 µL (thresholded LAMP-OSD reactions) to 5 µL (standard LAMP-OSD reactions) volume. These included either zero to 10^9^ orders of magnitude copies/reaction of synthetic 23S DNA, indicated dilutions of lab-cultivated *E. coli* or recombinant *E. coli* expressing *E. faecalis* 23S rDNA fragment, zero to few thousand colony forming units (CFU) of lab-cultivated *E. faecalis*, or 2.5 µL of filtration-enriched environmental water sample prepared as described below in the Methods subsection titled ‘Environmental sample collection and analysis’. Some LAMP-OSD reactions were seeded with *E. faecalis* 23S rRNA transcripts instead of DNA templates. Transcripts were generated by *in vitro* transcription followed by DNase I treatment using the HiScribe^®^ T7 High Yield RNA Synthesis Kit (NEB) and the manufacturer’s protocols. RNA was purified using RNeasy columns (Qiagen) and used as templates in LAMP-OSD reactions either before or after treatment with RNase cocktail (Thermo Fisher Scientific). Negative control reactions lacking specific templates were included each time a LAMP-OSD assay was performed.

All LAMP-OSD assays were performed at least in triplicate by incubation at 65 °C for 60 min and accumulation of OSD fluorescence was either measured in real-time using a Roche LightCycler^®^ 96 real-time PCR machine (Basel, Switzerland) or imaged at endpoint using a ChemiDoc MP imaging system (Hercules, California) or a blue light transilluminator and cellphone camera.

### 23S TaqMan qPCR assay

PCR primers and TaqMan probe described in the US EPA Method 1611 *E. faecalis* 23S qPCR assay: Enterococci in Water by TaqMan^®^ Quantitative Polymerase Chain Reaction (qPCR) Assay were used for qPCR analyses (Fig. S2) performed using the Luna^®^ Universal probe qPCR Master Mix according to the manufacturer’s instructions (NEB) (USEPA, 2012). Each sample was analyzed using three technical replicates of the qPCR reactions that were incubated at 95 °C for 10 min prior to 45 cycles of denaturation at 95 °C for 15 s and annealing and extension at 60 °C for 2 min. TaqMan probe fluorescence was measured in the FAM channel during extension. The qPCR efficiency and standard curve are depicted in Fig. S2.

### Environmental sample collection and analysis

Environmental water samples were collected from the urbanized and degraded Waller Creek (Ging, Lee & Silva, 1996; Clamann et al., 2019). Samples were collected at the intersection of Dean Keeton St. and San Jacinto Blvd in Austin, Texas, USA (GPS coordinates DD 30.28857, −97.73382; Table S2). Prior to sample collection, sampling bottles were acid-washed with 10% HCl to minimize contamination. Prior to sampling from a shaded, flowing section of the creek bottles were rinsed three times with creek water that was shaken and discarded downstream. Samples were transported and maintained on ice and processed within 3 h of collection.

For enterococci nucleic acid analyses, 400 mL of creek water samples were filtered using a vacuum manifold and MF-Millipore, 0.22 µm mixed cellulose esters (MCE) Membrane qPCR filters (Ref. No. GSWP04700). Following filtration samples were crudely processed using the Filter, Heat, Spin protocol described in Pham et al. (2025) with some modifications. In brief, sterile tweezers were used to fold and transfer the filters into 1.5 mL microcentrifuge tubes. 500 µL of AE buffer (10 mM Tris-Cl, 0.5 mM EDTA, pH of 9.0) was added to the tubes to fully submerge the filters. The tubes were then placed in a 95 °C heat block for 10 min followed by vortex mixing for 10 s and room temperature centrifugation at 12,000 rpm (9678 RCF) for 2 min. Some 250 µL of these processed samples in AE buffer were aliquoted into 1.5 mL tubes and stored at −20 °C until LAMP or qPCR analysis.

For analyzing enterococci colony forming units (CFU), five mL of the water samples were added to 100 mL of 1X phosphate buffered saline (137 mM NaCl, 2.7 mM KCl, 10 mM Na_2_HPO_4_, and 1.8 mM KH_2_PO_4_, pH 7.4) and filtered through white gridded 47 mm MCE S-Pak^®^ membrane filters of 0.45 µm pore size (Ref. No. HAWG047S6), then washed with approximately 20 mL of fresh PBS. The filters were then transferred to membrane *Enterococcus* Indoxyl-β-D-Glucoside (mEI) Agar plates (Moltox^®^ Molecular Toxicology, Boone, NC, USA) using sterile tweezers. The plates were inverted and incubated at 41 °C ± 0.5 °C for 24 h ± 2 h. Subsequently, colonies that produced a blue halo were presumptively identified as enterococci and their recorded count was used to calculate the CFU/mL of the water samples.

### Image and statistical analysis

Images of LAMP-OSD reactions were analyzed for signal intensity using browser-based ImageJ.JS (https://ij.imjoy.io/) and its Gels analysis tool according to the ImageJ user guide. Three or more independent biological replicates were performed for all experiments. Microsoft Excel built-in functions were used to calculate sample mean, sample standard deviation, and confidence intervals using Student’s t-distribution.

## Results

### Design of a 6-primer LAMP-OSD assay for enterococci

The enterococci LAMP reaction first reported by Martzy et al. (2017) utilized real-time fluorescence measurement with a DNA binding dye to evaluate the presence or absence of enterococci in environmental samples. The primers target the same region of the 23S rRNA gene as the EPA enterococci TaqMan qPCR test and were shown to detect a diverse range of *Enterococcus* species. However, DNA binding dyes cannot distinguish spurious from real amplification products, and the opening of LAMP reaction tubes for readout, *via* methods such as gel electrophoresis, can potentially lead to cross-contamination.

**Figure 1:**
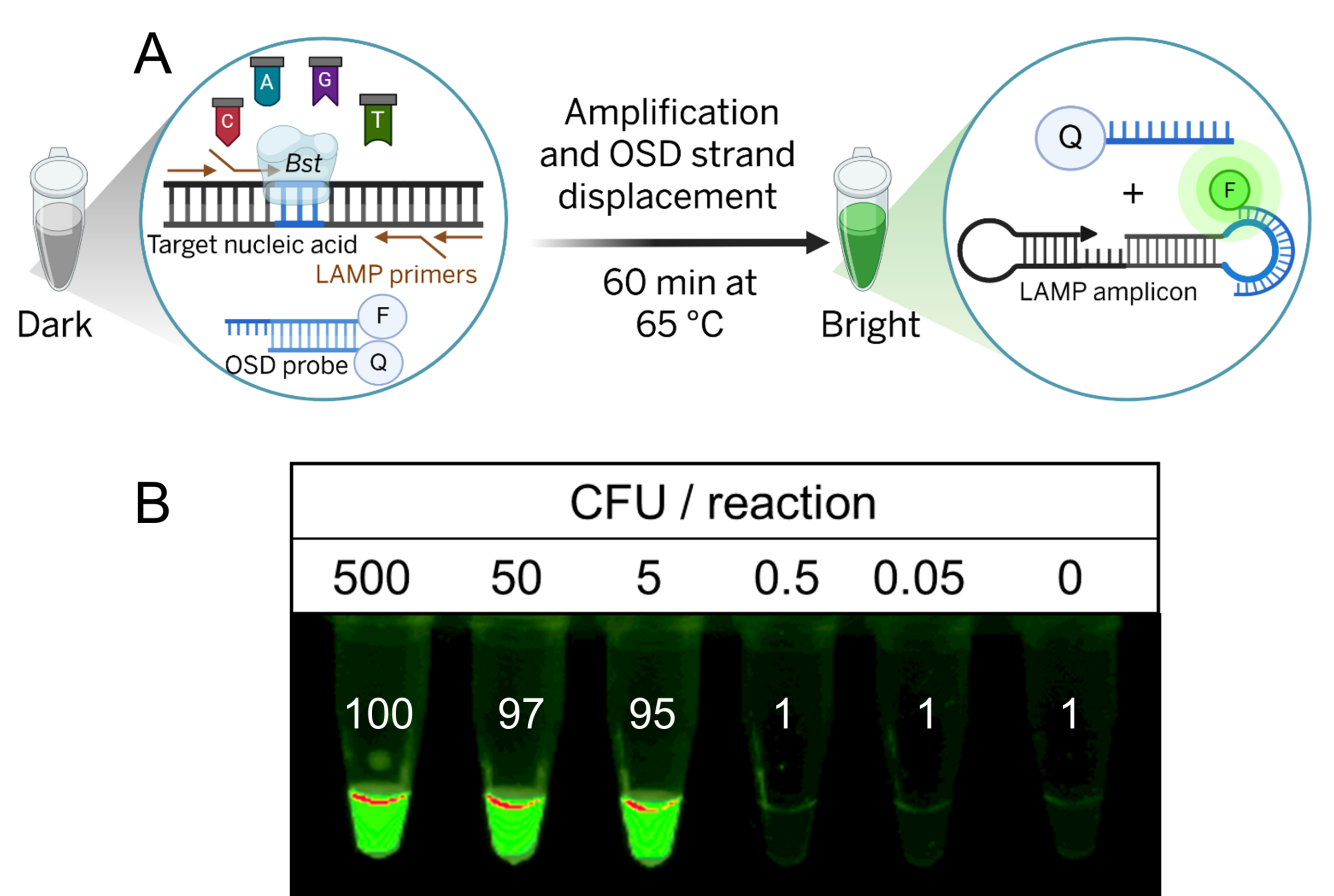
Visual LAMP-OSD analysis of lab-cultivated *Enterococcus faecalis* bacteria. (A) LAMP-OSD reaction schematic (Created in BioRender. B, S. (2025) https://BioRender.com/kgdg491). ‘*Bst*’ indicates the LAMP DNA polymerase while nucleotides are depicted as ‘A’, ‘T’, ‘C’, and ‘G’-labeled flags. In the pre-amplification LAMP-OSD reaction (‘Dark’), the gray shaded ‘Q’ and ‘F’ notations on the OSD hemiduplex represent the quencher moiety and the fluorophore in its quenched state, respectively. In the post-amplification LAMP-OSD reaction (‘Bright’), accumulation of OSD signal in response to correct LAMP amplicons is depicted as a fluorescent (green shaded ‘F’) amplicon-bound OSD long strand that has separated from the shorter quencher-labeled complementary strand. (B) Image of endpoint OSD fluorescence in LAMP-OSD assays following amplification of indicated colony forming units of log-phase *E. faecalis* is depicted. The numbers inset in each tube indicate the ImageJ measurements of the intensity of the imaged OSD signal relative to the endpoint OSD signal in reactions containing 500 CFU of *E. faecalis*. Relative intensity of ≥ 50 indicates a positive (‘bright’) LAMP-OSD reaction containing detectable *E. faecalis* while relative intensities similar to the negative control lacking specific templates are considered ‘dark’ implying undetectable *E. faecalis*. Data shown is representative of triplicate analysis.

We therefore sought to make enterococci detection more convenient and reliable using closed-tube visual observation, while also enabling a specific, semi-quantitative format, which should prove valuable for monitoring water quality against standard quantitative contamination thresholds. The first step towards converting the enterococci LAMP assay into a semi-quantitative visual assay was the development of a hemiduplex OSD probe for readout (Table S1 and Fig. 1), similar to how we have previously been able to specifically detect LAMP amplicons (Bhadra, Esteve-Gasent & Ellington, 2023; Bhadra et al., 2018a; Bhadra et al., 2021b; Jiang et al., 2015). The designed probe could interact with the amplicon loop sequence between the B1 and B2c primer recognition regions and upon strand displacement separate a fluor:quencher pair. Since this region of the loop was also occupied by the loop B primer, the OSD probe was designed to have the same polarity as the loop B primer to prevent primer:probe hybridization.

To validate that the OSD probe could be used for enterococci LAMP visual readout without compromising time-to-result, detection limit, or specificity (Martzy et al., 2017), LAMP assays containing all six LAMP primers (FIP, BIP, F3, B3, loop B and loop F) and the designed OSD probe were seeded with different numbers of colony forming units of lab-cultivated, logarithm phase *Enterococcus faecalis*. A specific bright OSD signal was observed in endpoint LAMP reactions containing as few as five CFU of *E. faecalis* (Fig. 1), confirming that LAMP-OSD visual ‘yes/no’ detection was feasible without the need for extraction and purification of nucleic acids from the cultured bacteria. LAMP-OSD assays containing fewer than five CFU of *E. faecalis* remained as dark as the negative control reactions lacking specific templates, suggesting that these analyte levels were below the detection limit of this LAMP-OSD reaction. Similarly, LAMP-OSD assays seeded with *E. coli* instead of *E. faecalis* remained dark (Fig. S3). Similar detection levels were observed in two additional biological replicate experiments where LAMP-OSD analysis of 10-fold dilution series of fresh bacterial cultures yielded bright fluorescence with as few as four and 10 CFU of *E. faecalis*, respectively, while negative controls remained dark (Fig. S3). These results suggest a 100% sensitivity of detection at mean CFU/reaction of 6.3 ± 2.1 (95% CI [1.2–11.5], *n* = 3). The overlap of the OSD long strand and the loop B primer did not compromise assay sensitivity (Fig. S4), providing a new dimension for probe and primer design. Furthermore, sequence alignment analysis performed using the Basic Local Alignment Search Tool (BLAST) confirmed that the LAMP amplicon region recognized by the OSD probe demonstrates 100% sequence conservation across multiple *Enterococcus* species (Table S3). This indicates that the enterococci LAMP-OSD test should be able to detect varied environmental isolates, including *E. faecalis, E. faecium*, *E. hirae*, *E. casseliflavus*, *E. durans*, and *E. raffinosus*, though detection levels might vary across different *Enterococcus* species (Zaheer et al., 2020).

### Development of signal thresholded LAMP-OSD for enterococci semi-quantitation using a single visual readout

**Figure 2:**
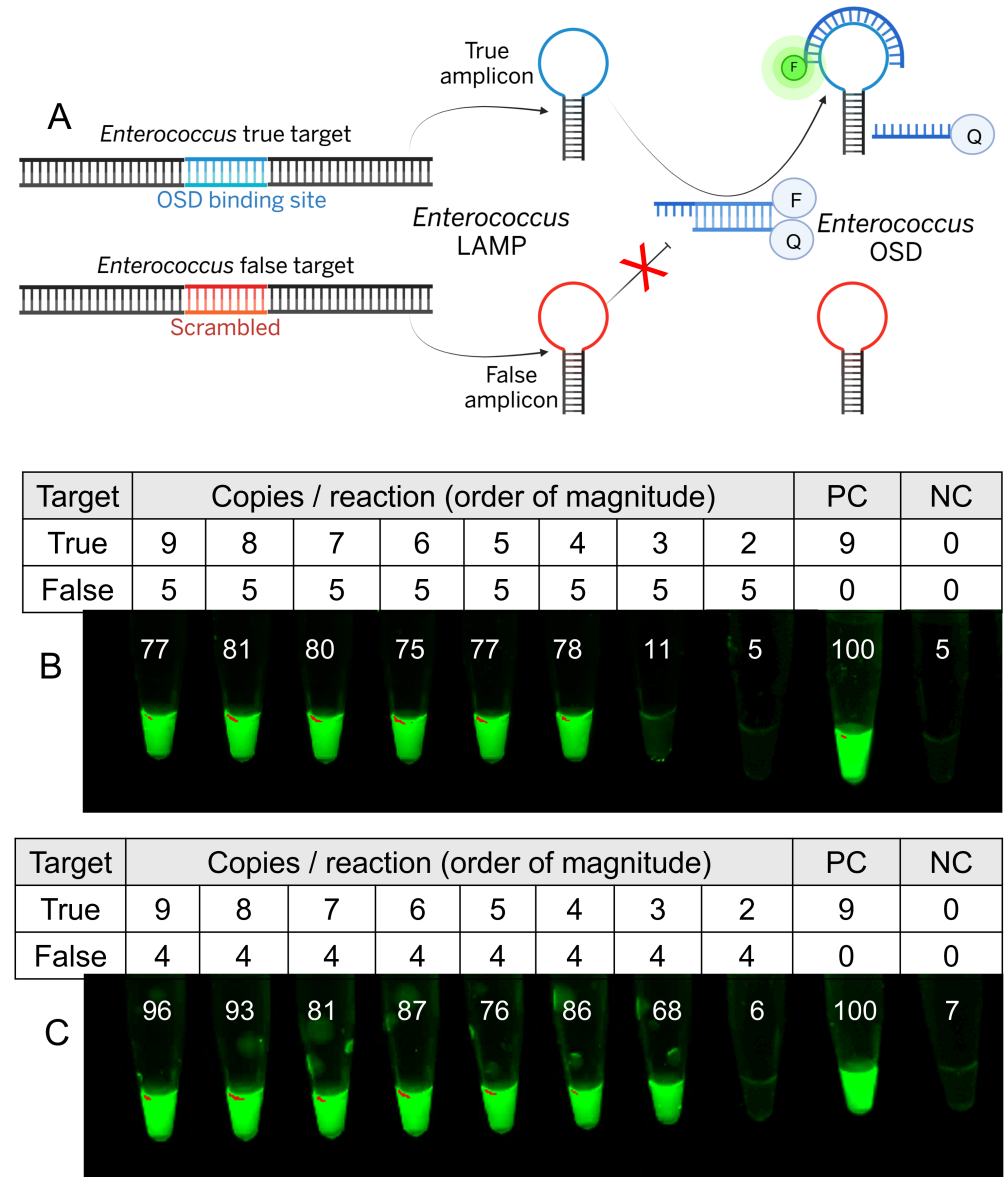
Signal thresholded LAMP-OSD analysis of synthetic *Enterococcus* DNA. (A) Schematic depicting signal thresholded LAMP-OSD. F and Q represent fluorophore (gray: quenched; green: unquenched) and quencher, respectively (Created in BioRender. B, S. (2025) https://BioRender.com/kgdg491). (B and C) Images of endpoint OSD fluorescence in a panel of thresholded *Enterococcus* LAMP-OSD assays following amplification of indicated amounts of synthetic true and false targets. The numbers inset in each tube indicate the intensity of its imaged endpoint OSD signal relative to the positive control (PC; seeded only with true targets) endpoint OSD signal measured using ImageJ. Relative intensity of ≥ 50 indicates a positive (‘bright’) reaction signifying that amplification of the indicated true target copies had outcompeted false target amplification while relative intensities similar to the negative control (NC; lacking any templates) are considered ‘dark’ indicating that amplification of true targets in these reactions had not outcompeted false target amplification. Results representative of at least triplicate experiments are depicted.

We have previously developed semi-quantitative methods for evaluating LAMP-OSD by seeding reactions with competitor (false target, FT) amplicons (Fig. 2A) (Jiang et al., 2017). The false target is identical to the LAMP true target (TT) except that the region recognized by the OSD reporter is scrambled. Consequently, it competes with the true target for amplification resources without generating a commensurate OSD fluorescence response and establishes a copy number threshold that the true target must exceed in order to generate a visible signal.

A synthetic ‘false’ target for the enterococci 23S rRNA gene was introduced into two sets of three biological replicate LAMP-OSD tests at 10^5^ (Fig. 2B and Fig. S5) or 10^4^ (Fig. 2C and Fig. S5) copies/reaction. Given that each bacterium would contain multiple copies of the 23S rRNA gene and transcripts (Suttner et al., 2021), these amounts of false targets were chosen to analyze the ability of thresholded LAMP-OSD to distinguish relatively low levels of contamination. These reactions were then seeded with ‘true’ synthetic DNA templates and visual OSD fluorescence was observed at amplification endpoint. In LAMP-OSD reactions containing on the order of 10^5^ false target copies, bright OSD fluorescence (≥50 relative units compared to the positive control with only true templates) was observed only in tubes containing at least 10^4^ or more copies of true targets, whereas tubes containing fewer true targets remained dark (<50 relative units). Meanwhile, LAMP-OSD assays thresholded with ten-fold lower amounts of false targets (on the order of 10^4^ copies/reaction) produced bright OSD fluorescence when amplifying at least 10^3^ or more copies of true targets. In both cases the ratio of false targets / true targets that allowed validated detection was found to be about 10 (inflection ratio = max(FT/TT); Fig. 2 and Fig. S5). These results suggest that presence or absence of OSD signal in thresholded LAMP-OSD reactions indicates whether the amount of true templates in the reaction is above (bright fluorescence) or below (dark) the chosen false target threshold. Therefore, testing a sample with a panel of LAMP-OSD reactions containing 10-fold incremental amounts of enterococci false targets should allow semi-quantitative order-of-magnitude determination of true target copies in the sample.

**Figure 3:**
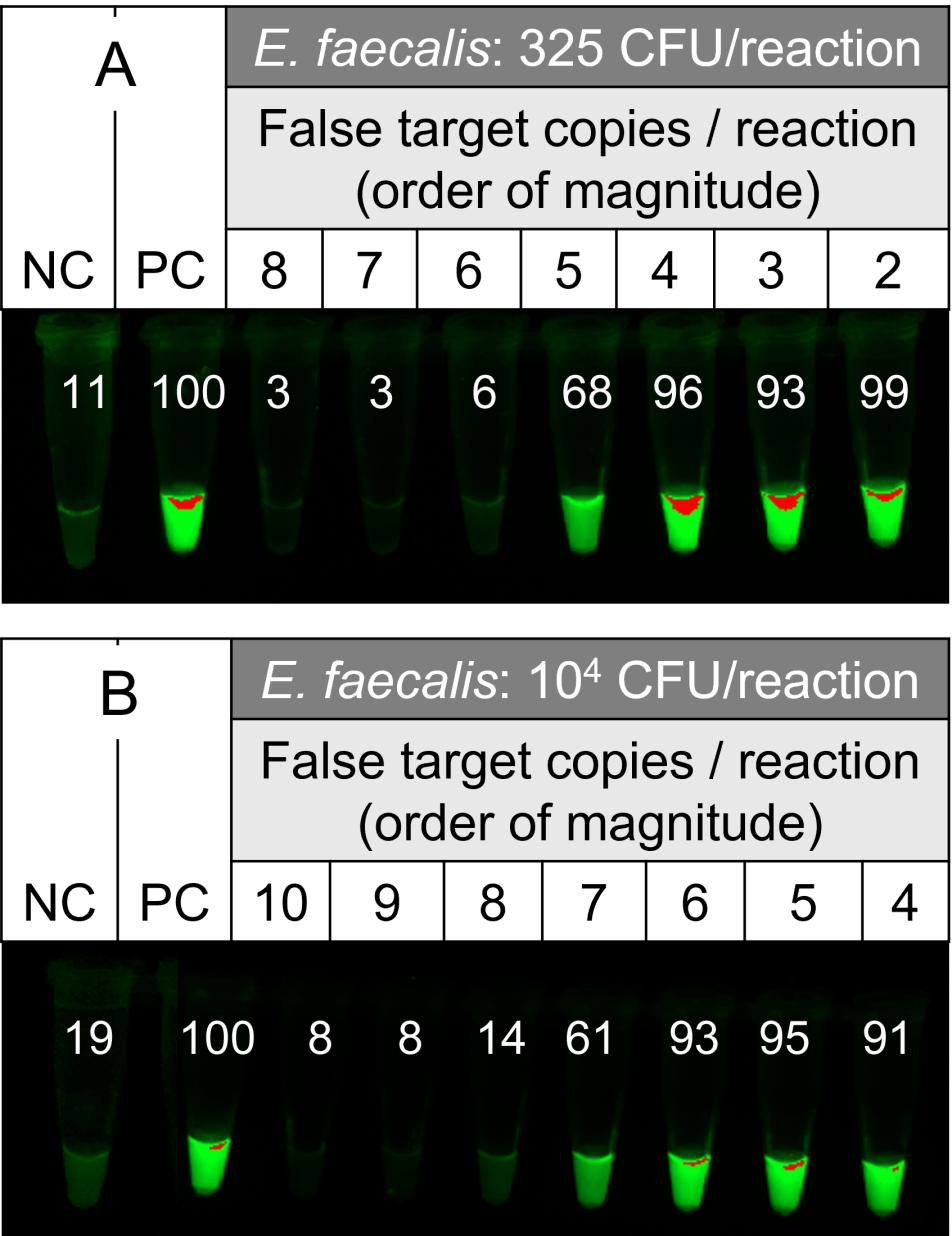
Signal thresholded LAMP-OSD analysis of *Enterococcus faecalis* bacteria. (A and B) Images of endpoint OSD fluorescence in two biological replicate panels of thresholded LAMP-OSD assays following amplification of indicated colony forming units (CFU) of lab-cultivated *E. faecalis* and log_10 copies/reaction of synthetic false targets. The numbers inset in each tube indicate the intensity of its imaged endpoint OSD signal relative to the positive control (PC) endpoint OSD signal measured using ImageJ. Relative intensity of ≥ 50 indicates a positive (‘bright’) reaction where amplification of the indicated CFUs of *E. faecalis* had outcompeted false target amplification while relative intensities similar to the negative control (NC) are considered ‘dark’ where amplification of the amount of *E. faecalis* in the reaction had not outcompeted false target amplification. NC, negative control lacking any templates; PC, positive control containing synthetic true target templates.

To determine whether thresholding would work with crude bacterial samples, four biological replicate assays containing on the order of 10^2^ or 10^4^ CFU / reaction of *E. faecalis* (rather than pure DNA) were spiked with varying amounts of false targets on an order of magnitude scale. At amplification endpoint, assays containing about 10^2^ CFU of *E. faecalis* produced bright OSD fluorescence only in reactions where false target thresholds were ≤10^5^(Fig. 3A and Fig. S6). Meanwhile, 10^4^ CFU of *E. faecalis* produced bright fluorescence only when the false target threshold was set at ≤10^7^copies (Fig. 3B and Fig. S6). These results suggest that different amounts of *Enterococcus* bacteria can be distinguished on an order of magnitude scale. The inflection ratio (max(FT/CFU)) for thresholded LAMP-OSD determination of *Enterococcus* colony forming units was found to be about 10^3^, and is consistent with the notion that each bacteria has multiple DNA and RNA copies of the 23S rRNA target sequence (since *Bst* DNA polymerase has considerable reverse transcriptase activity, allowing it to amplify both DNA and RNA targets during LAMP; Fig. S7) (Kim et al., 2023; Karpinets et al., 2006; Shi et al., 2015).

### Application of thresholded LAMP-OSD for semi-quantitation of enterococci in environmental samples

Having demonstrated the ability of thresholded LAMP-OSD to distinguish lab-cultivated *Enterococcus* amounts on the log_10_ scale, we hypothesized that the thresholded LAMP-OSD assay would be able to distinguish between different levels of enterococci contamination even in environmental water samples. To evaluate this hypothesis, three biological replicate experiments were performed using independent 400 mL of freshwater samples that were collected on different days from a local creek (Table S2). Microbial contaminants in the samples were concentrated on filter paper discs according to protocols detailed in the Methods section and heat-lysed in 500 µL buffer prior to analysis of 2.5 µL aliquots by TaqMan qPCR and thresholded LAMP-OSD tests. Since Waller Creek is reported to frequently contain high levels of fecal bacterial contamination (Olivera Perez & Reichler, 2025; Texas Commission on Environmental Quality (TCEQ), 2025), enterococci colony forming units were also measured by cultivating microbial contaminants filtered from five mL of water on mEI agar plates.

**Figure 4:**
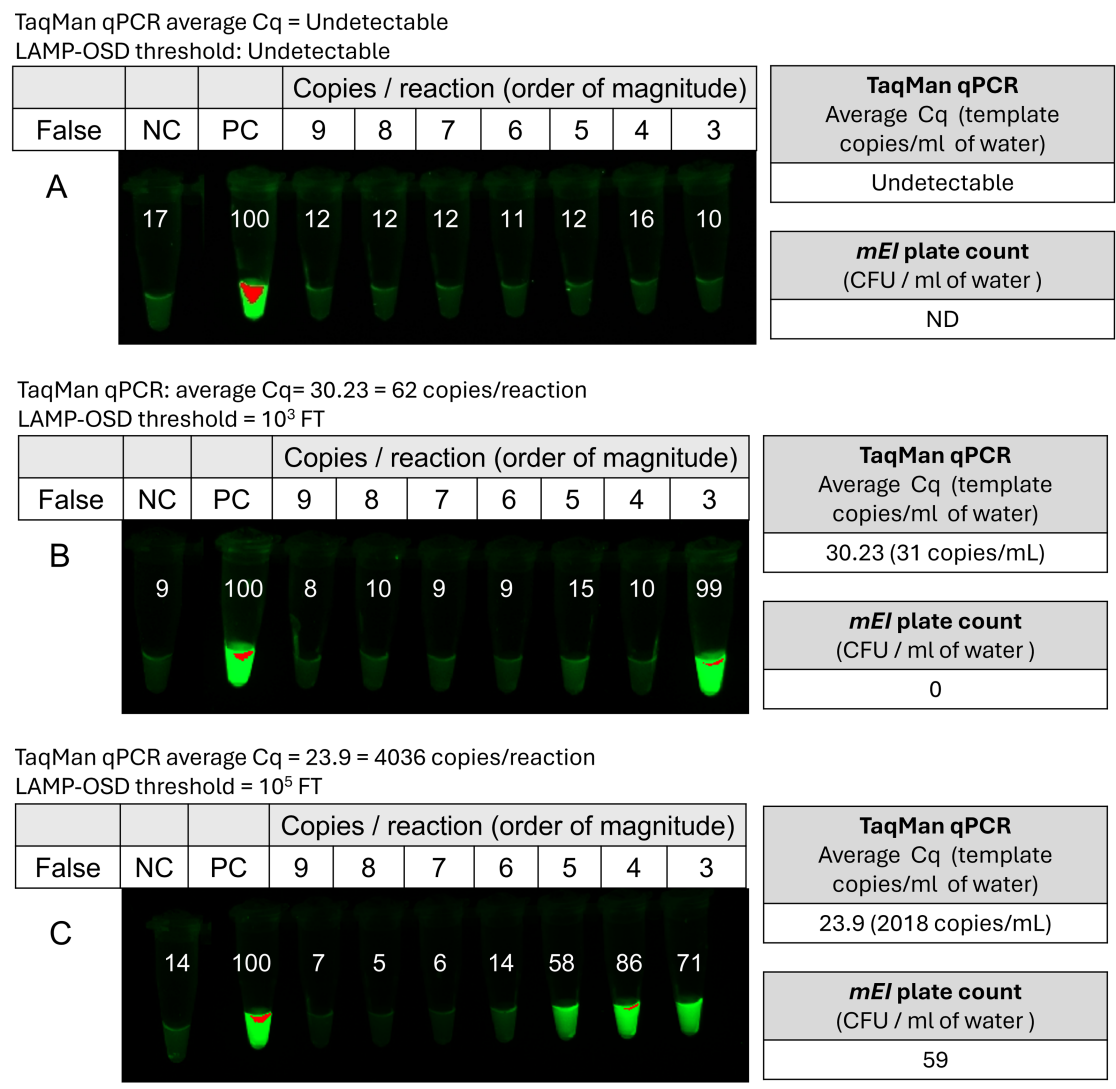
Signal thresholded LAMP-OSD analysis of *Enterococcus* bacteria in environmental freshwater samples. Images of endpoint OSD fluorescence in a panel of thresholded *Enterococcus* LAMP-OSD assays of three different water samples (A, B, and C) collected from Waller creek on different days are depicted. The OSD signal in the LAMP assays was thresholded with indicated log_10 copies/reaction of synthetic false targets. The numbers inset in each tube indicate the intensity of its imaged endpoint OSD signal relative to the positive control (PC) endpoint OSD signal measured using ImageJ. Relative intensity of ≥ 50 indicates a positive (‘bright’) reaction signifying that LAMP amplification of enterococci enriched from the water sample had outcompeted the synthetic false target amplification threshold while relative intensities similar to the negative control (NC) are considered ‘dark’ implying that the reaction lacked sufficient enterococci to outcompete the amplification of false targets. NC, negative control lacking any templates; PC, positive control containing synthetic true target templates. Cq values were converted to copies/reaction and copies/ml of original water sample by using a standard curve (Fig. S2). LAMP-OSD threshold was calculated as the maximum false target (FT) amount that allowed bright OSD fluorescence as observed at amplification endpoint (Fig. S2). ND, not determined.

Water samples in which enterococci could not be detected by TaqMan qPCR (*n* = 3) also failed to generate any OSD fluorescence in thresholded LAMP-OSD assays (Fig. 4A). Water samples in which qPCR yielded a measurable average Cq (quantification cycle) value of 30.2 ± 0.1 (*n* = 3) (62 templates/reaction) (but zero CFU/mL (MF) likely because enterococci were cultured from only five mL water sample) produced bright endpoint OSD fluorescence in LAMP assays thresholded with 10^3^ ± 0 (*n* = 2) orders of magnitude of false target copies. Assays thresholded with higher amounts of false targets remained as dark as the negative control lacking specific templates (Fig. 4B). Water samples containing about two orders of magnitude higher levels of enterococci contamination with an average qPCR Cq of 23.9 ± 0.09 (*n* = 3) (4,036 templates/reaction) and 59 CFU/mL, produced observable endpoint OSD fluorescence in LAMP assays thresholded with as much as 10^5^ ± 0 (*n* = 2) orders of magnitude of false target copies (Fig. 4C). These results demonstrate that in the tested water samples thresholded LAMP-OSD test outcomes were positively correlated with both enterococci enrichment culture and nucleic acid copies quantified by qPCR (Fig. S8). Water samples with higher numbers of enterococci and their nucleic acids also required proportionately higher amount of false targets to significantly diminish the OSD signal. Consequently, the magnitude of the LAMP-OSD false target threshold that allowed visual detection of bright OSD signal (≥50% intensity of positive control) was qualitatively on par with qPCR Cq values in identifying different (low, medium, or high) degrees of enterococci contamination in the tested environmental water samples, while requiring virtually no sample preparation.

## Discussion

Environmental surveillance of microorganisms is an indispensable tool for assuring public health and safeguarding ecological integrity (Santarpia et al., 2023; Thompson et al., 2020; Roseng et al., 2024; Ramljak et al., 2024). While culture-based detection of viable organisms or PCR-based nucleic acid tests are often gold standards for microbial monitoring (Lappan et al., 2021; Davis et al., 2022), their long time-to-result and/or relatively high cost often make widespread and same-day actionable use difficult (Griffith & Weisberg, 2011). Execution simplicity, speed, open-source protocols, and readily available reagents have led to the emergence of LAMP as a promising alternative for environmental surveillance (Moehling et al., 2021; Becherer et al., 2020; Wong et al., 2018). A LAMP assay that targets the enterococci 23S rRNA gene was recently reported to have comparable specificity and sensitivity to the US EPA enterococci qPCR test for surface water analysis (Martzy et al., 2017; USEPA, 2012). However, this LAMP assay, which was read using a DNA intercalating dye, cannot estimate the amount of enterococci in a sample. This is a drawback because determinations of recreational water quality for fecal contamination in fresh and marine water rely on quantitative thresholds, such as a geometric mean threshold of 35 colony forming units (CFU)/100 mL for culture-based detection methods and ≥ 1,000 calibrator cell equivalents (CCE)/100 mL for enterococci qPCR (Saleem et al., 2023; EPA, 2012).

We have now re-engineered this assay as a thresholded LAMP-OSD test for semi-quantitatively assessing the level of enterococcal contamination in environmental water. The threshold is set by including known amounts of false targets in LAMP-OSD reactions to compete with true targets for amplification resources but without generating OSD signal. Consequently, the amounts of purified DNA templates or enterococcal bacteria can be semi-quantitatively estimated as 10-fold or 1,000-fold lower, respectively, than the highest false target amounts that allow bright OSD signal. The higher apparent threshold sensitivity of enterococci is consistent with the presence of multiple 23S rDNA and rRNA copies per bacteria and the ability of LAMP-OSD to utilize both nucleic acids as templates. When tested with a small set of environmental water samples, this assay performed on par with qPCR and was able to readily distinguish water samples that contained different levels of enterococci contamination with no non-specific signal. There was clearly a correspondence between enterococci plate counts, the detection of DNA copies by PCR (62, 4036), and nucleic acid semi-quantitation by LAMP-OSD (10^3^ and 10^5^ false target threshold, respectively), indicating that LAMP-OSD has the potential to provide actionable answers over a real-world dynamic range. Previous studies have shown that subjective assessment of test results by users have proven reliable, including in self-testing and clinical diagnostics (Anand et al., 2024). That said, field ready instruments capable of evaluating OSD fluorescence are readily available if there is a need to minimize any potential user error (Bhadra & Ellington, 2022; Ding et al., 2019).

Although the current study is restricted to the analysis of a small set of flowing freshwater samples collected from the Waller creek, we have previously demonstrated the compatibility of the LAMP-OSD system with rapid analysis of a variety of contaminated water sources that might inhibit enzymatic reactions, such as chlorinated water, water containing humic acids, and sea water. The simple expedient of rinsing the microbial filter once with distilled water post sample filtration was found to be sufficient for eliminating potential inhibitors and allowing direct LAMP-OSD analysis of filter-retained bacteria without further nucleic acid purification. Therefore, with further validation using a broader set of environmental samples, this approach might prove generalizable for microbial water quality monitoring.

Enterococci qPCR can provide absolute quantitation (detecting as few as 27 enterococci/100 mL sample within 2.5 to 4 h), but also requires complex instrumentation (Haugland et al., 2005). The feasibility of LAMP-based quantitative microbial source tracking of fecal contamination has also been demonstrated (Xu et al., 2025), but would similarly rely on real-time qPCR machines to measure template concentration-dependent changes in time-to-LAMP signal, making field implementation difficult and potentially as expensive as qPCR. In contrast, the thresholded LAMP-OSD assay needs only one hour of incubation at a single temperature to detect as few as five CFU of enterococci per reaction, and semi-quantitative readout is accomplished *via* simple visual inspection of OSD fluorescence at assay endpoint. While the gold standard culture-based IDEXX Enteroalert can detect one CFU/100 mL sample and may be used to analyze enterococci in smaller sample volumes, it typically takes 24 h and requires specialized equipment and consumables. By maintaining low infrastructure requirement and ease of simple endpoint visual readout, LAMP-OSD retains the potential for remote field use and real-time water quality monitoring while facilitating rapid identification of enterococci contamination above preset actionable false target thresholds. Furthermore, the sequence-dependent readout *via* OSD probes may also mitigate the spurious signal and reduced concordance with qPCR often observed during qualitative monitoring of fecal contamination with LAMP (Wang et al., 2023).

The implementation of semi-quantitative LAMP-OSD can be further enhanced by using freeze-dried reaction mixes and low-cost remote chemical or battery-operated heaters (Jiang et al., 2018; Halphen et al., 2024; Singleton et al., 2014). Using the market price of assay components, the cost per reaction of LAMP-OSD is $1.5 that includes $0.16 of primers and $0.28 of OSD probes (IDT; both costs calculated after adjusting for lower than synthesis scale yields), $0.26 of dNTPs (NEB), $0.1 of betaine (Sigma), and $0.7 for Bst 2.0 (NEB). Although this price is significantly lower than that of qualitative colorimetric LAMP (NEB; $2 per reaction without primers) the cost of thresholded LAMP-OSD may be relatively higher when used as a panel of reactions with different thresholds. Operating costs may be further reduced by using robust engineered LAMP enzymes and low-cost production platforms (Paik, Bhadra & Ellington, 2022; Paik et al., 2021; Bhadra et al., 2022; Bhadra et al., 2021a) and LAMP reactions optimized for lower temperature and therefore instrumentation needs (Cai et al., 2018). Indeed, the gold standard culture-based IDEXX Enteroalert test and the TaqMan qPCR nucleic acid test, cost about $15 to $30 per sample without factoring in the tens of thousands of dollars of initial instrument set up cost and the potentially recurring maintenance and laboratory infrastructure costs (Griffith & Weisberg, 2011).

## Conclusions

This study reports the development and application of an easy-to-use rapid nucleic acid assay system, termed thresholded LAMP-OSD, for semi-quantitation of enterococci in environmental water by direct isothermal amplification of filter-concentrated heat-lysed bacteria and visual endpoint readout of presence or absence of fluorescence. The assay uses known amounts of false LAMP targets that can be amplified but cannot bind the OSD probes to threshold enterococci 23S nucleic acid amplification such that OSD fluorescence is generated only when the false target concentration is at or below 1,000-fold of the bacterial count. In small scale tests with environmental freshwater samples, thresholded LAMP-OSD performed on par with qPCR and accurately distinguished water samples with low, medium, or high levels of enterococci contamination. Into the future, large scale performance metrics and generalizability of the thresholded enterococci LAMP-OSD assay, both for varied environmental sample types as well as environmental isolates of different *Enterococcus* species, can be assessed, along with our previously reported semi-quantitative assays for *E. coli* and the human fecal indicator HF183 (Jiang et al., 2018). These results support the possibility of using LAMP-OSD for low-cost acquisition of contamination data across a diversity of environmental and drinking water samples. Given their simplicity, the assays might be readily adapted by public health organizations, or even by citizen science groups.

##  Supplemental Information

10.7717/peerj.21310/supp-1 Supplemental Information 1 Oligonucleotide and template sequences used in the study

10.7717/peerj.21310/supp-2 Supplemental Information 2 Waller Creek sample collection conditions

10.7717/peerj.21310/supp-3 Supplemental Information 3 Alignment of the enterococci OSD probe with genomic sequences from varied *Enterococcus* species

10.7717/peerj.21310/supp-4 Supplemental Information 4 * Enterococcus faecalis* template sequence annotated with LAMP and qPCR primers and probes

10.7717/peerj.21310/supp-5 Supplemental Information 5 Parameters for estimation of template amounts by TaqMan qPCR and thresholded LAMP-OSD (A) *Enterococcus* TaqMan qPCR standard curve. Triplicate amplification kinetics of indicated copies of synthetic *Enterococcus* DNA templates. NC: negative control lacking specific templates. (B) Standard curve analysis of amplification data using the LightCycler Abs Quant analysis with template copies per reaction on the X-axis and the corresponding qPCR Cq values on the Y-axis. (C) Equations derived from the standard curve analysis used for calculation of copies/reaction and copies/ml of a water sample using its qPCR Cq data. (D) Metric for calculation of thresholded LAMP-OSD false target threshold.

10.7717/peerj.21310/supp-6 Supplemental Information 6 Validation of enterococci LAMP-OSD assay detection sensitivity and specificity (A) LAMP-OSD analysis of *Escherichia coli* before and after transformation with an *Enterococcus* 23S rRNA encoding plasmid. Image of endpoint OSD fluorescence in 6-primer LAMP-OSD reactions following amplification of indicated dilutions of logarithm phase cultures of *E. coli* bacteria that were either untransformed (lacking *Enterococcus* sequences) or were expressing a plasmid encoding the *Enterococcus* 23S rRNA sequence. The numbers inset in each tube indicate the ImageJ measured intensity of its imaged endpoint OSD signal relative to the positive control endpoint OSD signal (reaction with 10 -4 dilution factor *Enterococcus* 23S rRNA plasmid transformed bacteria). Relative intensity of ≥ 50 indicates a positive (‘bright’) reaction containing detectable amounts of *E. coli* transformants while relative intensities similar to the negative control are considered ‘dark’ implying undetectable levels of *Enterococcus* sequences. (B and C) Two additional biological replicates of visual LAMP-OSD analysis of lab-cultivated *Enterococcus faecalis* bacteria. Images of endpoint OSD fluorescence in LAMP-OSD assays following amplification of indicated colony forming units of log-phase *E. faecalis* are depicted. NC, negative control lacking templates; EC, specificity control seeded with *E. coli*.

10.7717/peerj.21310/supp-7 Supplemental Information 7 5-primer versus 6-primer LAMP analysis of *Enterococcus* synthetic DNA templates Real-time measurement of *Enterococcus* LAMP amplicon accumulation using OSD probe fluorescence and either 5-primer (A) or 6-primer (B) mediated amplification in LAMP reactions containing indicated copies of synthetic *Enterococcus* 23S DNA templates is depicted. NTC: no template control. Representative results of at least triplicate experiments are depicted.

10.7717/peerj.21310/supp-8 Supplemental Information 8 Replicates of signal thresholded LAMP-OSD analysis of synthetic *Enterococcus* DNA Images of endpoint OSD fluorescence in two biological replicates each of thresholded *Enterococcus* LAMP-OSD assays following competitive amplification of synthetic DNA false targets at 10 4 (A) or 10 5 (B) copies/reaction and the indicated amounts of true *Enterococcus* 23S synthetic DNA. The numbers inset in each tube indicate the intensity of its imaged endpoint OSD signal relative to the positive control (PC; seeded only with true targets) endpoint OSD signal measured using ImageJ. Relative intensity of ≥ 50 indicates a positive (‘bright’) reaction signifying that amplification of the indicated true target copies had outcompeted false target amplification while relative intensities similar to the negative control (NC) are considered ‘dark’ indicating that amplification of true targets in these reactions could not outcompete false target amplification.

10.7717/peerj.21310/supp-9 Supplemental Information 9 Replicates of signal thresholded LAMP-OSD analysis of *Enterococcus faecalis* bacteria Images of endpoint OSD fluorescence in panels of thresholded LAMP-OSD assays following amplification of indicated colony forming units (CFU) of lab-cultivated *E. faecalis* and log10 copies/reaction of synthetic false target DNA. NC: negative control lacking any templates; PC: positive control containing only synthetic true template DNA. The numbers inset in each tube indicate the intensity of its imaged endpoint OSD signal relative to the positive control (PC) endpoint OSD signal measured using ImageJ. Relative intensity of ≥ 50 indicates a positive (‘bright’) reaction where amplification of the indicated CFUs of *E. faecalis* had outcompeted false target amplification while relative intensities similar to the negative control (NC) are considered ‘dark’ where amplification of the amount of *E. faecalis* in the reaction could not outcompete false target amplification.

10.7717/peerj.21310/supp-10 Supplemental Information 10 Detection of *Enterococcus* 23S rRNA transcripts using LAMP-OSD assay Indicated copies of 23S rRNA transcripts were analyzed using LAMP-OSD amplification before (solid lines) or after (dashed lines) treatment of the RNA templates with RNase cocktail for 1 h. Real-time measurement of OSD fluorescence accumulation in response to LAMP amplification of the *Enterococcus* RNA templates is depicted in panel A and endpoint imaging of OSD fluorescence in the same wells performed at LAMP reaction completion is depicted in panel B.

10.7717/peerj.21310/supp-11 Supplemental Information 11 Correlation between visually read thresholded LAMP-OSD and qPCR or plate cultures analyses of environmental water samples Scatter plots of data shown in Figure 4 depicting correlation between LAMP-OSD false target thresholds for visual detection of enterococci contamination level in environmental water and qPCR Cq values (A) or enterococci mEI plate counts (B).

10.7717/peerj.21310/supp-12 Supplemental Information 12 Raw data for qPCR standard curve

10.7717/peerj.21310/supp-13 Supplemental Information 13 Raw data for curves shown in Figure S7A

10.7717/peerj.21310/supp-14 Supplemental Information 14 Raw data for curves shown in Figure S4A

10.7717/peerj.21310/supp-15 Supplemental Information 15 Raw data for curves shown in Figure S4B

10.7717/peerj.21310/supp-16 Supplemental Information 16 Raw data for qPCR average Cq value shown in Figure 4A

10.7717/peerj.21310/supp-17 Supplemental Information 17 Raw data for qPCR average Cq value shown in Figure 4B

10.7717/peerj.21310/supp-18 Supplemental Information 18 Raw data for qPCR average Cq value shown in Figure 4C

10.7717/peerj.21310/supp-19 Supplemental Information 19 Image of mEI plate culture data reported in Figure 4B

10.7717/peerj.21310/supp-20 Supplemental Information 20 Image of mEI plate culture data reported in Figure 4C

10.7717/peerj.21310/supp-21 Supplemental Information 21 Raw image of photograph depicted in Table S2

## Additional Information and Declarations

### Data Availability

The following information was supplied regarding data availability: The raw data for qPCR analysis of samples, real-time LAMP-OSD, and environmental sample mEI plate cultures are available in the Supplemental Files.
