# Supplementary material for: Fieldable isothermal nucleic acid test for rapid semi-quantitative visual readout of enterococci in recreational waters
Source: PeerJ. 2026 May 22;14:e21310. doi: 10.7717/peerj.21310 (PMC13200618; doi:10.7717/peerj.21310)
Supplement: Supplemental Information 1 [file peerj-14-21310-s001.docx]

**Supplementary Table 1**. Oligonucleotide and template sequences used in the study.

| **Name** | **Sequence 5’ → 3’** | **References** |
| --- | --- | --- |
| qPCR fw primer | GAGAAATTCCAAACGAACTTG | USEPA^a^ |
| qPCR rev primer | CAGTGCTCTACCTCCATCATT | USEPA |
| qPCR TaqMan probe | /56-FAM/TGGTTCTCT/ZEN/CCGAAATAGCTTTAGGGCTA/3IABkFQ/ | USEPA |
| ENT-LAMP FIP (F1c-F2) | AACGTACGTGGGTTCGGTCCTTCTACCCATGTCCAGGTTGA | Martzy^b^ |
| ENT-LAMP BIP (B1-B2c) | GATGAGGTGTGGGTAGCGGAGACGAGGCTAGCCCTAAAGCT | Martzy |
| ENT-LAMP F3 | CGTAGACCCGAAACCATGTG | Martzy |
| ENT-LAMP B3 | ACAGTGCTCTACCTCCATCA | Martzy |
| ENT-LAMP LoopF | GTGCGTTTTACCGCACCT | Martzy |
| ENT-LAMP LoopB | AATTCCAAACGAACTTGGAGATAGC | Martzy |
| ENT-LAMP-OSD-FAM | /56-FAM/CGCAATTCCAAACGAACTTGGAGATAGCTGGTTCTCTCCG/3InvdT/ | This study |
| ENT-LAMP-OSD-Quencher | AGCTATCTCCAAGTTCGTTTGGAATTGCG/3IABkFQ/ | This study |
| *E. faecalis* 23S rDNA LAMP template | GGCCCCTAGTCCAAACAGTGCTCTACCTCCATCATTCTCAATTCCGAGGCTAGCCCTAAAGCTATTTCGGAGAGAACCAGCTATCTCCAAGTTCGTTTGGAATTTCTCCGCTACCCACACCTCATCCCCGCACTTTTCAACGTACGTGGGTTCGGTCCTCCAGTGCGTTTTACCGCACCTTCAACCTGGACATGGGTAGATCACATGGTTTCGGGTCTACGACTACATACTTATTCGCCCTATTCAGACTC | Martzy/EPA |
| False Template (Scrambled OSD binding region) | GGCCCCTAGTCCAAACAGTGCTCTACCTCCATCATTCTCAATTCCGAGGCTAGCCCTAAAGCTATTTCATCCTACTTGTCCTGGTATGAGCCAGTTGAAAGGAATCTCCGCTACCCACACCTCATCCCCGCACTTTTCAACGTACGTGGGTTCGGTCCTCCAGTGCGTTTTACCGCACCTTCAACCTGGACATGGGTAGATCACATGGTTTCGGGTCTACGACTACATACTTATTCGCCCTATTCAGACTC | This study |

^a^Method 1611: Enterococci in Water by TaqMan® Quantitative Polymerase Chain Reaction (qPCR) Assay. Available at https://www.epa.gov/sites/default/files/2015-08/documents/method_1611_2012.pdf.

^b^Martzy, R.; Kolm, C.; Brunner, K.; Mach, R. L.; Krska, R.; Šinkovec, H.; Sommer, R.; Farnleitner, A. H.; Reischer, G. H. A loop-mediated isothermal amplification (LAMP) assay for the rapid detection of Enterococcus spp. in water. *Water Res.* **2017**, *122*, 62-69. DOI: https://doi.org/10.1016/j.watres.2017.05.023.

/56-FAM/: 5’-end fluorescein; /3InvdT/: 3’-end inverted dT; /3IABkFQ/: 3’-end Iowa Black FQ quencher; /ZEN/: ZEN internal quencher.
