# Supplementary material for: Fieldable isothermal nucleic acid test for rapid semi-quantitative visual readout of enterococci in recreational waters
Source: PeerJ. 2026 May 22;14:e21310. doi: 10.7717/peerj.21310 (PMC13200618; doi:10.7717/peerj.21310)
Supplement: Supplemental Information 2 [file peerj-14-21310-s002.docx]

**Supplementary Table 2. Waller Creek sample collection conditions.**

| **Date** | ***Enterococcus* levels** | **Location** | **Conditions during sampling** | | | | | **Notes** |
| --- | --- | --- | --- | --- | --- | --- | --- | --- |
|  |  |  | **Time** | **Air temperature** | **Humidity** | **Wind** | **Amount of trash** |  |
| 10/2/2024 | Not detected | Creek Side Hall | 2:00 PM | 32 °C | 34% | 5 mph | Light, wrappers | No rains for several weeks |
| 11/6/2024 | High | Creek Side Hall | 9:30 AM | 18 °C | 61% | None | Light, wrappers & some plastic | Rain on 11/4 and 11/5, noted rushing water at sampling site |
| 1/23/2025 | Low | Creek Side Hall | 3:30 PM | 12 °C | 21% | 7 mph | Light, wrappers & a can | Light snow two days prior to sampling |

**
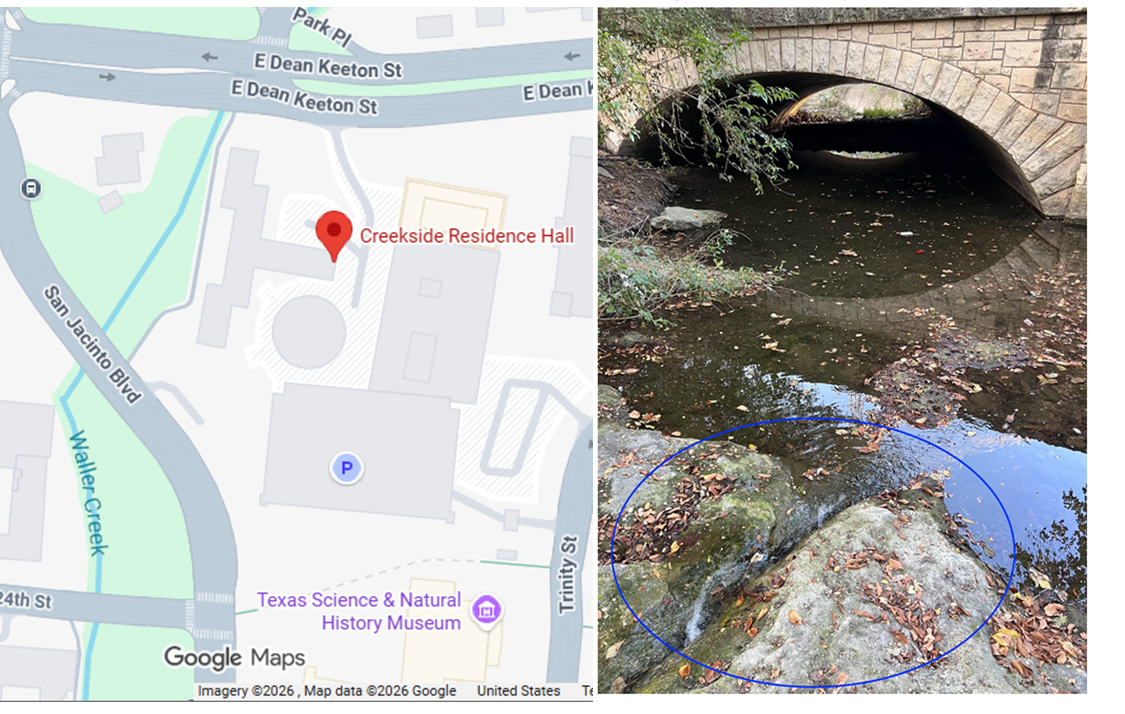
**

**Location map (Map data ©2026 Google)** **and image of Creek Side Hall sampling site.**
