# Supplementary material for: Fieldable isothermal nucleic acid test for rapid semi-quantitative visual readout of enterococci in recreational waters
Source: PeerJ. 2026 May 22;14:e21310. doi: 10.7717/peerj.21310 (PMC13200618; doi:10.7717/peerj.21310)
Supplement: Supplemental Information 3 [file peerj-14-21310-s003.docx]

**Supplementary Table 3. Alignment of the enterococci OSD probe with genomic sequences from varied *Enterococcus* species.**

| **Description^a^** | **Scientific Name** | **Max Score** | **Total Score** | **Query Cover** | **E value** | **Per. ident** | **Acc. Len** | **Accession** |
| --- | --- | --- | --- | --- | --- | --- | --- | --- |
| Enterococcus hirae strain S46-3 chromosome, complete genome | Enterococcus hirae | 68 | 408 | 100% | 5.00E-12 | 100 | 2759528 | [CP088187.1](https://www.ncbi.nlm.nih.gov/nucleotide/CP088187.1?report=genbank&log$=nucltop&blast_rank=1&RID=J9MXDF3J014) |
| Enterococcus hirae strain 726p chromosome, complete genome | Enterococcus hirae | 68 | 408 | 100% | 5.00E-12 | 100 | 2676653 | [CP170115.1](https://www.ncbi.nlm.nih.gov/nucleotide/CP170115.1?report=genbank&log$=nucltop&blast_rank=2&RID=J9MXDF3J014) |
| Enterococcus hirae strain FDAARGOS_234 chromosome | Enterococcus hirae | 68 | 408 | 100% | 5.00E-12 | 100 | 2845651 | [CP023011.2](https://www.ncbi.nlm.nih.gov/nucleotide/CP023011.2?report=genbank&log$=nucltop&blast_rank=3&RID=J9MXDF3J014) |
| Enterococcus hirae ATCC 9790 isolate Enterococcus hirae | Enterococcus hirae ATCC 9790 | 68 | 408 | 100% | 5.00E-12 | 100 | 2780723 | [LR994474.1](https://www.ncbi.nlm.nih.gov/nucleotide/LR994474.1?report=genbank&log$=nucltop&blast_rank=4&RID=J9MXDF3J014) |
| Enterococcus hirae 23S rRNA gene, strain LMG 6399 (T) | Enterococcus hirae | 68 | 68 | 100% | 5.00E-12 | 100 | 2914 | [AJ295309.1](https://www.ncbi.nlm.nih.gov/nucleotide/AJ295309.1?report=genbank&log$=nucltop&blast_rank=5&RID=J9MXDF3J014) |
| Enterococcus gallinarum strain EGM181 chromosome | Enterococcus gallinarum | 68 | 340 | 100% | 2.00E-12 | 100 | 3726097 | CP050485.1 |
| Enterococcus gallinarum strain K205-4a chromosome | Enterococcus gallinarum | 68 | 272 | 100% | 2.00E-12 | 100 | 2669879 | CP116510.1 |
| Enterococcus gallinarum strain fecal_iso1 chromosome | Enterococcus gallinarum | 68 | 340 | 100% | 2.00E-12 | 100 | 3445024 | CP078505.1 |
| Enterococcus gallinarum strain 933p chromosome | Enterococcus gallinarum | 68 | 340 | 100% | 2.00E-12 | 100 | 3264692 | CP170114.1 |
| Enterococcus gallinarum strain 574p chromosome | Enterococcus gallinarum | 68 | 340 | 100% | 2.00E-12 | 100 | 3284599 | CP169563.1 |
| Enterococcus casseliflavus strain FDAARGOS_1121 chromosome | Enterococcus casseliflavus | 68 | 340 | 100% | 3.00E-12 | 100 | 3511859 | CP068128.1 |
| Enterococcus casseliflavus strain E1 | Enterococcus casseliflavus | 68 | 68 | 100% | 3.00E-12 | 100 | 4266 | MK322659.1 |
| Enterococcus flavescens 23S rRNA gene, strain LMG 13518 (T) | Enterococcus casseliflavus | 68 | 68 | 100% | 3.00E-12 | 100 | 2915 | AJ295307.1 |
| Enterococcus casseliflavus strain Dec0527 chromosome | Enterococcus casseliflavus | 68 | 340 | 100% | 3.00E-12 | 100 | 3345060 | CP141640.1 |
| Enterococcus casseliflavus strain ASE4 chromosome | Enterococcus casseliflavus | 68 | 340 | 100% | 3.00E-12 | 100 | 3662325 | CP119393.1 |
| Enterococcus raffinosus strain E11 | Enterococcus raffinosus | 68 | 68 | 100% | 1.00E-12 | 100 | 4258 | [MK322666.1](https://www.ncbi.nlm.nih.gov/nucleotide/MK322666.1?report=genbank&log$=nucltop&blast_rank=1&RID=J9N5KKR1016) |
| Enterococcus raffinosus strain HG-5 chromosome | Enterococcus raffinosus | 68 | 340 | 100% | 1.00E-12 | 100 | 3258457 | [CP104392.1](https://www.ncbi.nlm.nih.gov/nucleotide/CP104392.1?report=genbank&log$=nucltop&blast_rank=2&RID=J9N5KKR1016) |
| Enterococcus raffinosus strain ATCC 49464 chromosome | Enterococcus raffinosus | 68 | 408 | 100% | 1.00E-12 | 100 | 3274514 | [CP104762.1](https://www.ncbi.nlm.nih.gov/nucleotide/CP104762.1?report=genbank&log$=nucltop&blast_rank=3&RID=J9N5KKR1016) |
| Enterococcus raffinosus strain F162_2 chromosome | Enterococcus raffinosus | 68 | 408 | 100% | 1.00E-12 | 100 | 3032004 | [CP072888.1](https://www.ncbi.nlm.nih.gov/nucleotide/CP072888.1?report=genbank&log$=nucltop&blast_rank=4&RID=J9N5KKR1016) |
| Enterococcus raffinosus strain Er676 chromosome | Enterococcus raffinosus | 68 | 408 | 100% | 1.00E-12 | 100 | 3200986 | [CP104764.1](https://www.ncbi.nlm.nih.gov/nucleotide/CP104764.1?report=genbank&log$=nucltop&blast_rank=5&RID=J9N5KKR1016) |
| Enterococcus avium strain Bang_SAM2.39.S1 chromosome | Enterococcus avium | 68 | 408 | 100% | 7.00E-13 | 100 | 3098697 | [CP145094.1](https://www.ncbi.nlm.nih.gov/nucleotide/CP145094.1?report=genbank&log$=nucltop&blast_rank=1&RID=J9N812AD014) |
| Enterococcus avium strain ATCC 14025 23S ribosomal RNA gene | Enterococcus avium ATCC 14025 | 68 | 68 | 100% | 7.00E-13 | 100 | 2541 | [AY116900.1](https://www.ncbi.nlm.nih.gov/nucleotide/AY116900.1?report=genbank&log$=nucltop&blast_rank=2&RID=J9N812AD014) |
| Enterococcus avium strain E16 | Enterococcus avium | 68 | 68 | 100% | 7.00E-13 | 100 | 4273 | [MK322658.1](https://www.ncbi.nlm.nih.gov/nucleotide/MK322658.1?report=genbank&log$=nucltop&blast_rank=3&RID=J9N812AD014) |
| Enterococcus avium 23S rRNA gene, strain LMG 10744 (T) | Enterococcus avium ATCC 14025 | 68 | 68 | 100% | 7.00E-13 | 100 | 2913 | [AJ295299.1](https://www.ncbi.nlm.nih.gov/nucleotide/AJ295299.1?report=genbank&log$=nucltop&blast_rank=4&RID=J9N812AD014) |
| Enterococcus avium strain 352 chromosome, complete genome | Enterococcus avium | 68 | 408 | 100% | 7.00E-13 | 100 | 4794392 | [CP034169.1](https://www.ncbi.nlm.nih.gov/nucleotide/CP034169.1?report=genbank&log$=nucltop&blast_rank=5&RID=J9N812AD014) |
| Enterococcus faecium strain C2159 chromosome | Enterococcus faecium | 71.8 | 430 | 100% | 1.00E-11 | 100 | 2895128 | [CP174843.1](https://www.ncbi.nlm.nih.gov/nucleotide/CP174843.1?report=genbank&log$=nucltop&blast_rank=1&RID=J9SDBJZD016) |
| Enterococcus faecium strain 16107_5 chromosome | Enterococcus faecium | 71.8 | 430 | 100% | 1.00E-11 | 100 | 2954299 | [CP175382.1](https://www.ncbi.nlm.nih.gov/nucleotide/CP175382.1?report=genbank&log$=nucltop&blast_rank=2&RID=J9SDBJZD016) |
| Enterococcus faecium strain VRE081 chromosome | Enterococcus faecium | 71.8 | 430 | 100% | 1.00E-11 | 100 | 2839148 | [CP111749.1](https://www.ncbi.nlm.nih.gov/nucleotide/CP111749.1?report=genbank&log$=nucltop&blast_rank=3&RID=J9SDBJZD016) |
| Enterococcus faecium strain VRE119 chromosome | Enterococcus faecium | 71.8 | 430 | 100% | 1.00E-11 | 100 | 2827148 | [CP112021.1](https://www.ncbi.nlm.nih.gov/nucleotide/CP112021.1?report=genbank&log$=nucltop&blast_rank=4&RID=J9SDBJZD016) |
| Enterococcus faecium strain VRE157 chromosome | Enterococcus faecium | 71.8 | 430 | 100% | 1.00E-11 | 100 | 2936843 | [CP112846.1](https://www.ncbi.nlm.nih.gov/nucleotide/CP112846.1?report=genbank&log$=nucltop&blast_rank=5&RID=J9SDBJZD016) |
| Enterococcus durans strain FDAARGOS 1437 chromosome | Enterococcus durans | 71.8 | 430 | 100% | 3.00E-13 | 100 | 2883932 | [CP077320.1](https://www.ncbi.nlm.nih.gov/nucleotide/CP077320.1?report=genbank&log$=nucltop&blast_rank=1&RID=J9SH85RJ016) |
| Enterococcus durans strain KLDS 6.0930, complete genome | Enterococcus durans | 71.8 | 430 | 100% | 3.00E-13 | 100 | 2867090 | [CP012384.1](https://www.ncbi.nlm.nih.gov/nucleotide/CP012384.1?report=genbank&log$=nucltop&blast_rank=2&RID=J9SH85RJ016) |
| Enterococcus durans strain S8-3 chromosome, complete genome | Enterococcus durans | 71.8 | 430 | 100% | 3.00E-13 | 100 | 3015822 | [CP088203.1](https://www.ncbi.nlm.nih.gov/nucleotide/CP088203.1?report=genbank&log$=nucltop&blast_rank=3&RID=J9SH85RJ016) |
| Enterococcus durans strain BT0150 chromosome | Enterococcus durans | 71.8 | 430 | 100% | 3.00E-13 | 100 | 3125355 | [CP186062.1](https://www.ncbi.nlm.nih.gov/nucleotide/CP186062.1?report=genbank&log$=nucltop&blast_rank=4&RID=J9SH85RJ016) |
| Enterococcus durans strain BT0139 chromosome | Enterococcus durans | 71.8 | 430 | 100% | 3.00E-13 | 100 | 2990689 | [CP186067.1](https://www.ncbi.nlm.nih.gov/nucleotide/CP186067.1?report=genbank&log$=nucltop&blast_rank=5&RID=J9SH85RJ016) |
| Enterococcus cecorum strain NCTC12421 genome assembly | Enterococcus cecorum | 71.8 | 502 | 100% | 3.00E-13 | 100 | 2421598 | [LS483306.1](https://www.ncbi.nlm.nih.gov/nucleotide/LS483306.1?report=genbank&log$=nucltop&blast_rank=1&RID=J9SK6HV2014) |
| Enterococcus cecorum strain SA2 genome | Enterococcus cecorum | 71.8 | 71.8 | 100% | 3.00E-13 | 100 | 2281990 | [CP010061.1](https://www.ncbi.nlm.nih.gov/nucleotide/CP010061.1?report=genbank&log$=nucltop&blast_rank=2&RID=J9SK6HV2014) |
| Enterococcus cecorum strain CE3 genome | Enterococcus cecorum | 71.8 | 71.8 | 100% | 3.00E-13 | 100 | 2372196 | [CP010063.1](https://www.ncbi.nlm.nih.gov/nucleotide/CP010063.1?report=genbank&log$=nucltop&blast_rank=3&RID=J9SK6HV2014) |
| Enterococcus cecorum strain SA1 genome | Enterococcus cecorum | 71.8 | 71.8 | 100% | 3.00E-13 | 100 | 2261297 | [CP010060.1](https://www.ncbi.nlm.nih.gov/nucleotide/CP010060.1?report=genbank&log$=nucltop&blast_rank=4&RID=J9SK6HV2014) |
| Enterococcus cecorum isolate CIRMBP-1228 genome assembly | Enterococcus cecorum | 71.8 | 502 | 100% | 3.00E-13 | 100 | 2799887 | [OX346405.1](https://www.ncbi.nlm.nih.gov/nucleotide/OX346405.1?report=genbank&log$=nucltop&blast_rank=5&RID=J9SK6HV2014) |
| Enterococcus casseliflavus strain FDAARGOS_1121 chromosome | Enterococcus casseliflavus | 71.8 | 359 | 100% | 3.00E-13 | 100 | 3511859 | [CP068128.1](https://www.ncbi.nlm.nih.gov/nucleotide/CP068128.1?report=genbank&log$=nucltop&blast_rank=1&RID=J9SMJ955016) |
| Enterococcus casseliflavus strain E1 16S ribosomal RNA gene | Enterococcus casseliflavus | 71.8 | 71.8 | 100% | 3.00E-13 | 100 | 4266 | [MK322659.1](https://www.ncbi.nlm.nih.gov/nucleotide/MK322659.1?report=genbank&log$=nucltop&blast_rank=2&RID=J9SMJ955016) |
| Enterococcus flavescens 23S rRNA gene, strain LMG 13518 (T) | Enterococcus casseliflavus | 71.8 | 71.8 | 100% | 3.00E-13 | 100 | 2915 | [AJ295307.1](https://www.ncbi.nlm.nih.gov/nucleotide/AJ295307.1?report=genbank&log$=nucltop&blast_rank=3&RID=J9SMJ955016) |
| Enterococcus casseliflavus strain Dec0527 chromosome | Enterococcus casseliflavus | 71.8 | 359 | 100% | 3.00E-13 | 100 | 3345060 | [CP141640.1](https://www.ncbi.nlm.nih.gov/nucleotide/CP141640.1?report=genbank&log$=nucltop&blast_rank=4&RID=J9SMJ955016) |
| Enterococcus casseliflavus strain ASE4 chromosome | Enterococcus casseliflavus | 71.8 | 359 | 100% | 3.00E-13 | 100 | 3662325 | [CP119393.1](https://www.ncbi.nlm.nih.gov/nucleotide/CP119393.1?report=genbank&log$=nucltop&blast_rank=5&RID=J9SMJ955016) |

^a^ Five hits per species resulting from sequence alignments of the OSD reporter and enterococci genomes performed using the NCBI BLAST tool are depicted.
