# Supplementary figures and images for: Fieldable isothermal nucleic acid test for rapid semi-quantitative visual readout of enterococci in recreational waters
Source: PeerJ. 2026 May 22;14:e21310. doi: 10.7717/peerj.21310 (PMC13200618; doi:10.7717/peerj.21310)

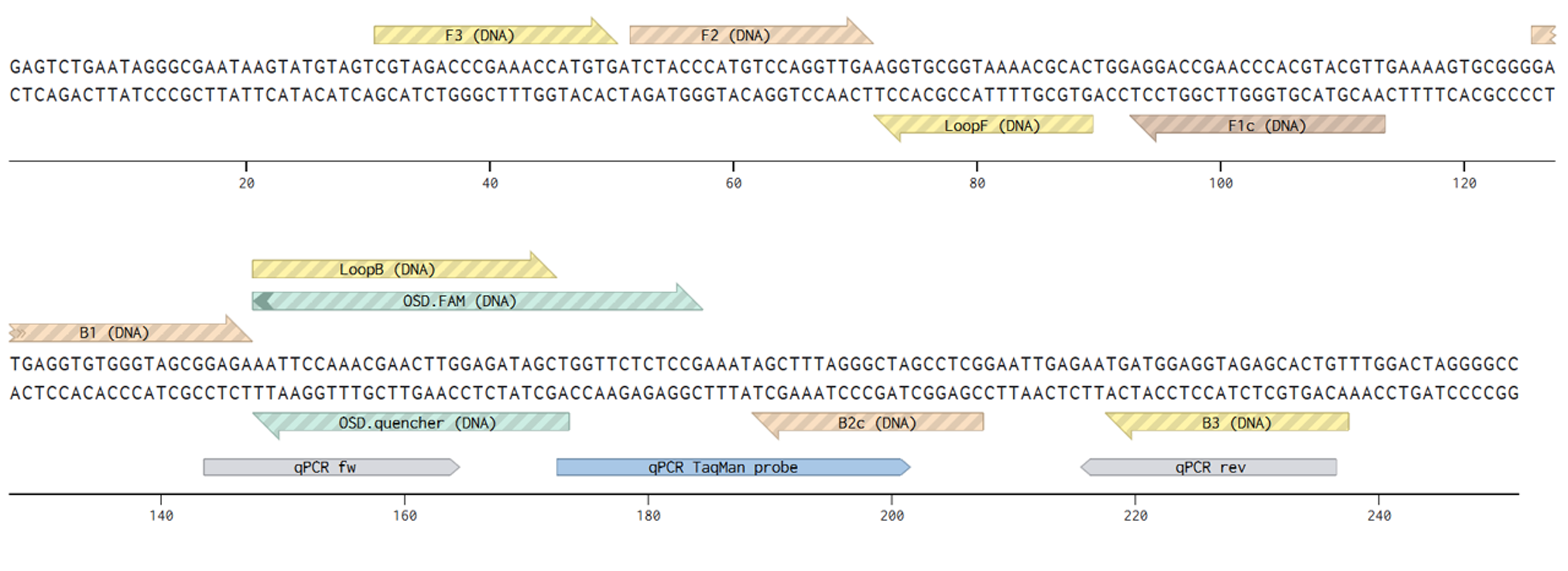

Supplement: Supplemental Information 4 [file peerj-14-21310-s004.png]

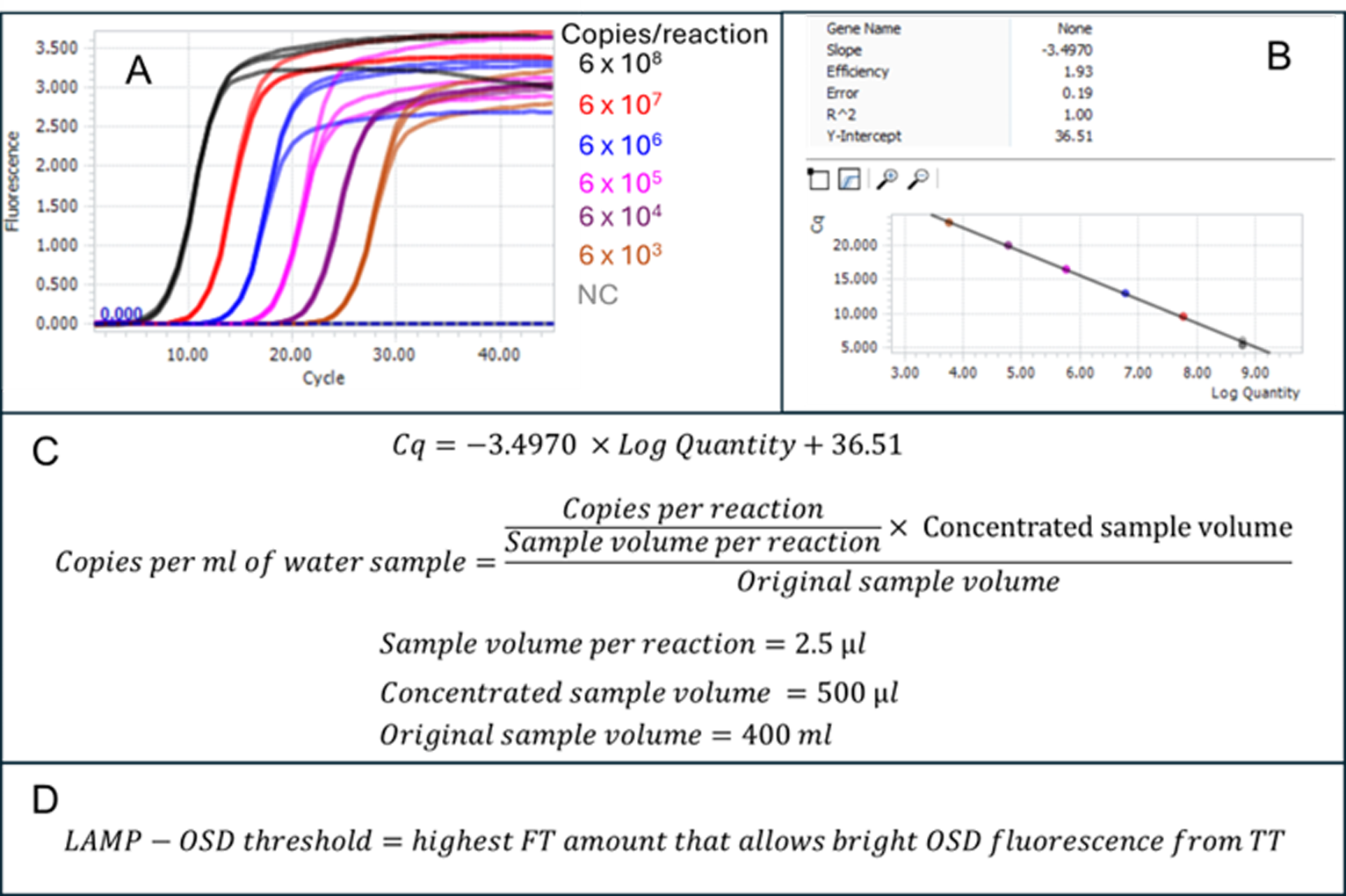

Supplement: Supplemental Information 5 — (A) Enterococcus TaqMan qPCR standard curve. Triplicate amplification kinetics of indicated copies of synthetic Enterococcus DNA templates. NC: negative control lacking specific templates. (B) Standard curve analysis of amplification data using the LightCycler Abs Quant analysis with template copies per reaction on the X-axis and the corresponding qPCR Cq values on the Y-axis. (C) Equations derived from the standard curve analysis used for calculation of copies/reaction and copies/ml of a water sample using its qPCR Cq data. (D) Metric for calculation of thresholded LAMP-OSD false target threshold. [file peerj-14-21310-s005.png]

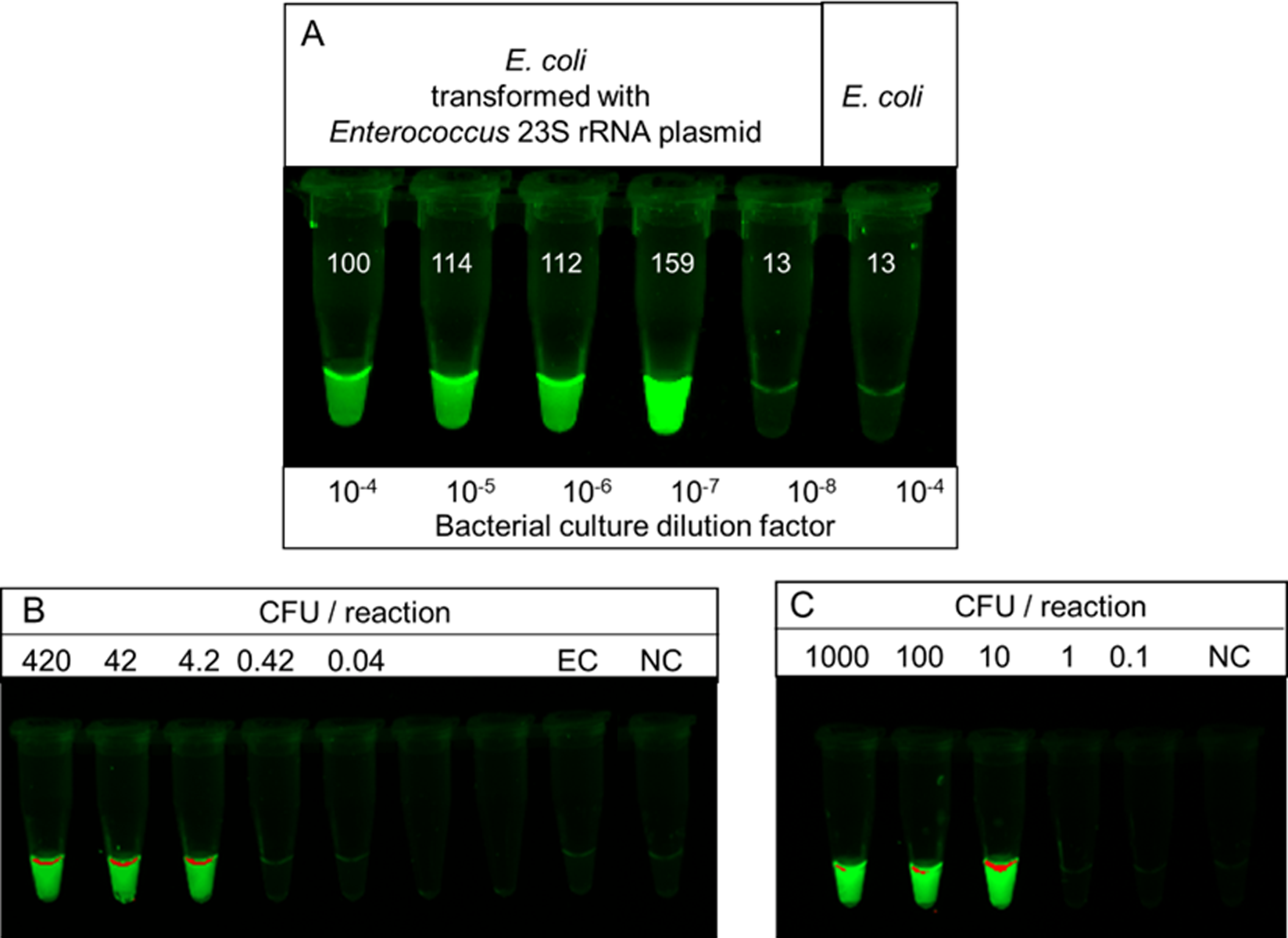

Supplement: Supplemental Information 6 — (A) LAMP-OSD analysis of Escherichia coli before and after transformation with an Enterococcus 23S rRNA encoding plasmid. Image of endpoint OSD fluorescence in 6-primer LAMP-OSD reactions following amplification of indicated dilutions of logarithm phase cultures of E. coli bacteria that were either untransformed (lacking Enterococcus sequences) or were expressing a plasmid encoding the Enterococcus 23S rRNA sequence. The numbers inset in each tube indicate the ImageJ measured intensity of its imaged endpoint OSD signal relative to the positive control endpoint OSD signal (reaction with 10 -4 dilution factor Enterococcus 23S rRNA plasmid transformed bacteria). Relative intensity of ≥ 50 indicates a positive (‘bright’) reaction containing detectable amounts of E. coli transformants while relative intensities similar to the negative control are considered ‘dark’ implying undetectable levels of Enterococcus sequences. (B and C) Two additional biological replicates of visual LAMP-OSD analysis of lab-cultivated Enterococcus faecalis bacteria. Images of endpoint OSD fluorescence in LAMP-OSD assays following amplification of indicated colony forming units of log-phase E. faecalis are depicted. NC, negative control lacking templates; EC, specificity control seeded with E. coli. [file peerj-14-21310-s006.png]

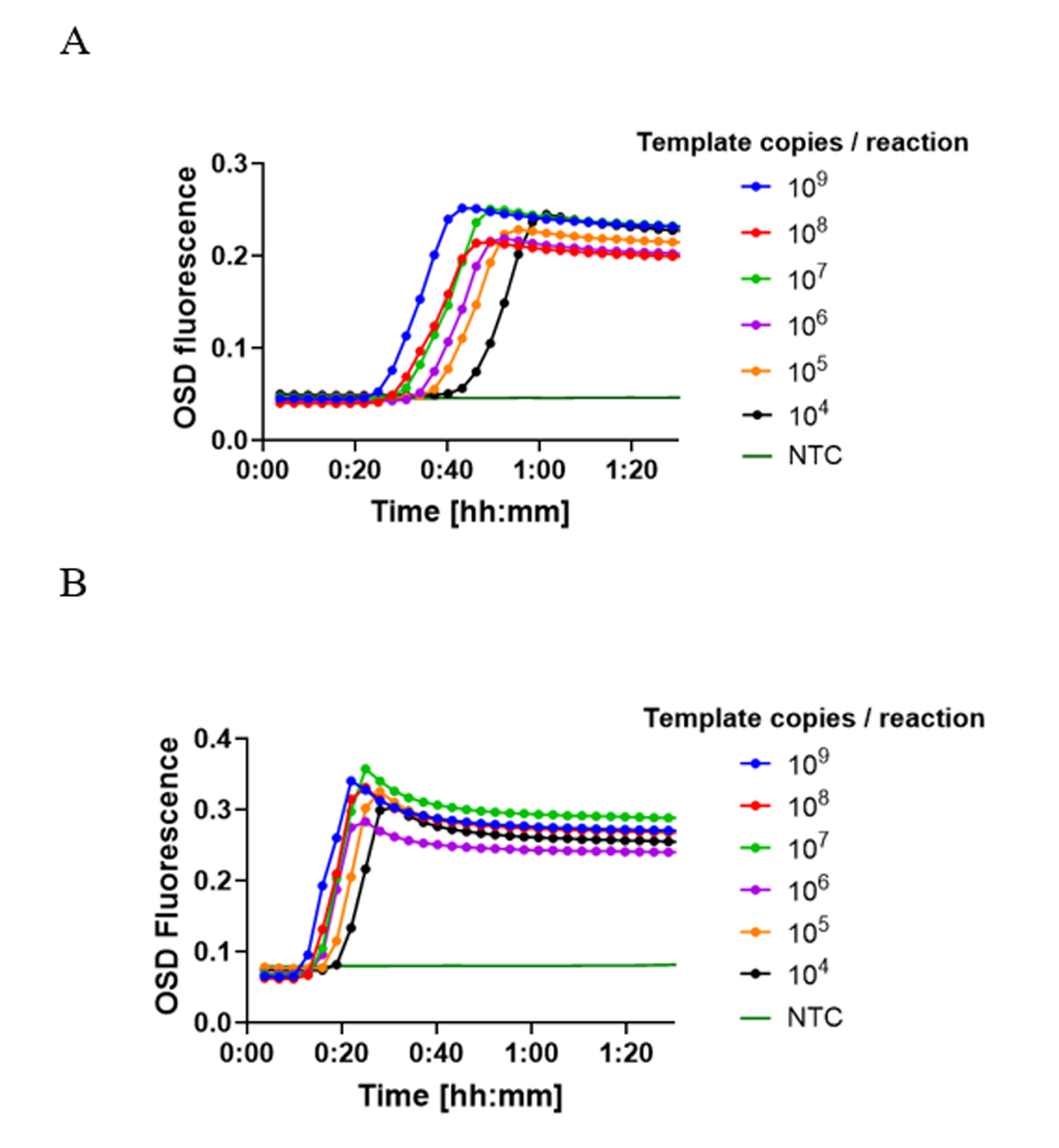

Supplement: Supplemental Information 7 — Real-time measurement of Enterococcus LAMP amplicon accumulation using OSD probe fluorescence and either 5-primer (A) or 6-primer (B) mediated amplification in LAMP reactions containing indicated copies of synthetic Enterococcus 23S DNA templates is depicted. NTC: no template control. Representative results of at least triplicate experiments are depicted. [file peerj-14-21310-s007.png]

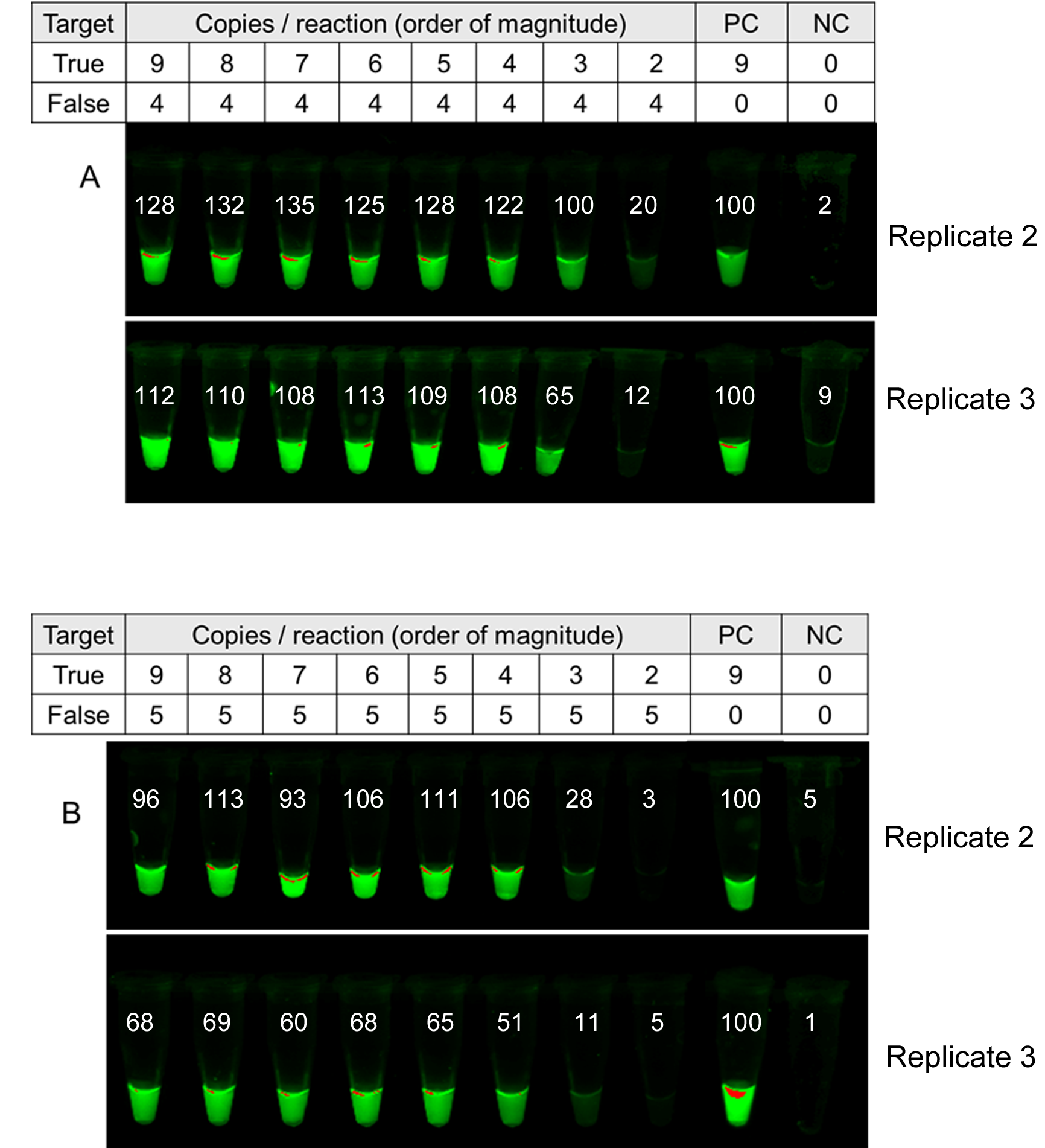

Supplement: Supplemental Information 8 — Images of endpoint OSD fluorescence in two biological replicates each of thresholded Enterococcus LAMP-OSD assays following competitive amplification of synthetic DNA false targets at 10 4 (A) or 10 5 (B) copies/reaction and the indicated amounts of true Enterococcus 23S synthetic DNA. The numbers inset in each tube indicate the intensity of its imaged endpoint OSD signal relative to the positive control (PC; seeded only with true targets) endpoint OSD signal measured using ImageJ. Relative intensity of ≥ 50 indicates a positive (‘bright’) reaction signifying that amplification of the indicated true target copies had outcompeted false target amplification while relative intensities similar to the negative control (NC) are considered ‘dark’ indicating that amplification of true targets in these reactions could not outcompete false target amplification. [file peerj-14-21310-s008.png]

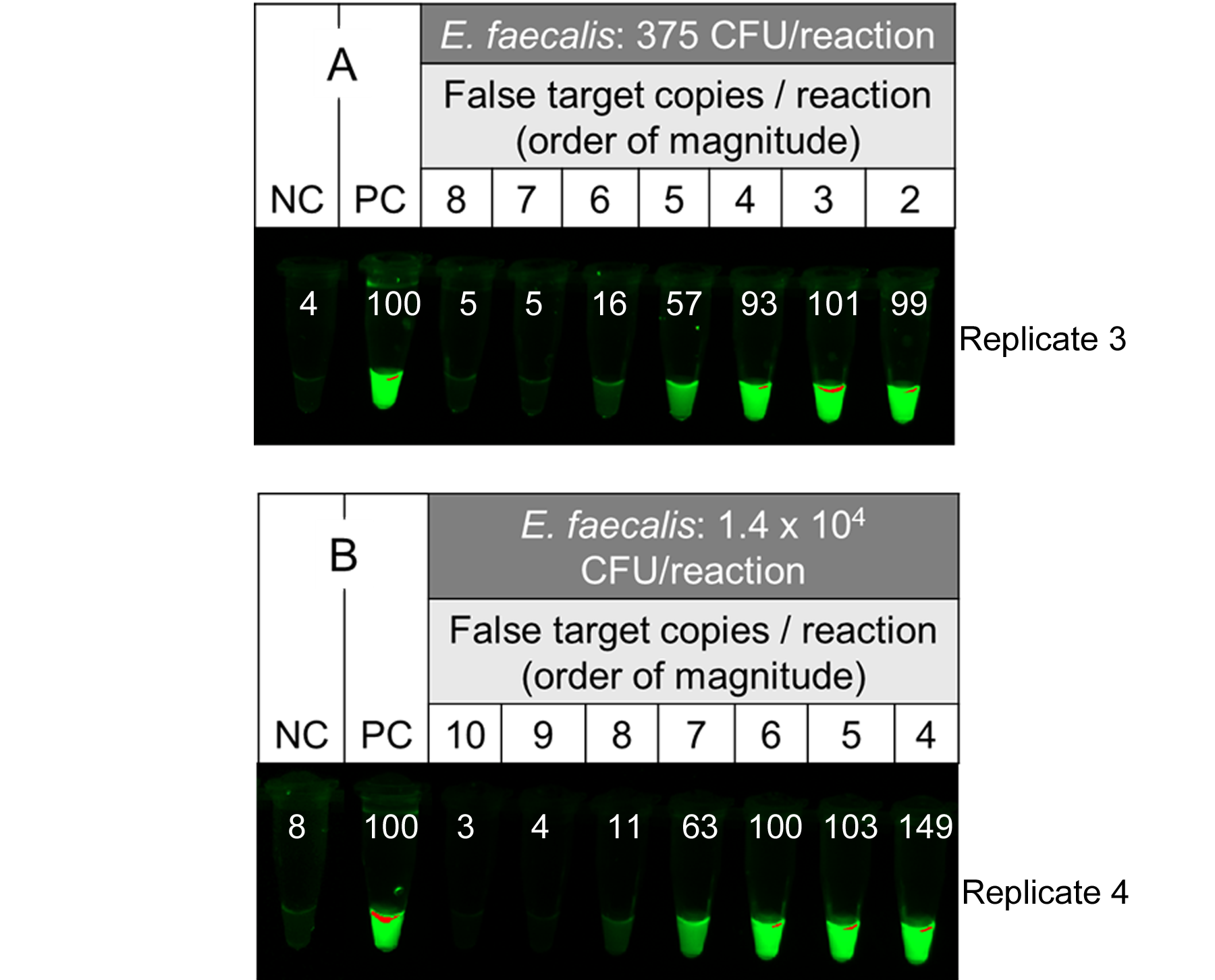

Supplement: Supplemental Information 9 — Images of endpoint OSD fluorescence in panels of thresholded LAMP-OSD assays following amplification of indicated colony forming units (CFU) of lab-cultivated E. faecalis and log10 copies/reaction of synthetic false target DNA. NC: negative control lacking any templates; PC: positive control containing only synthetic true template DNA. The numbers inset in each tube indicate the intensity of its imaged endpoint OSD signal relative to the positive control (PC) endpoint OSD signal measured using ImageJ. Relative intensity of ≥ 50 indicates a positive (‘bright’) reaction where amplification of the indicated CFUs of E. faecalis had outcompeted false target amplification while relative intensities similar to the negative control (NC) are considered ‘dark’ where amplification of the amount of E. faecalis in the reaction could not outcompete false target amplification. [file peerj-14-21310-s009.png]

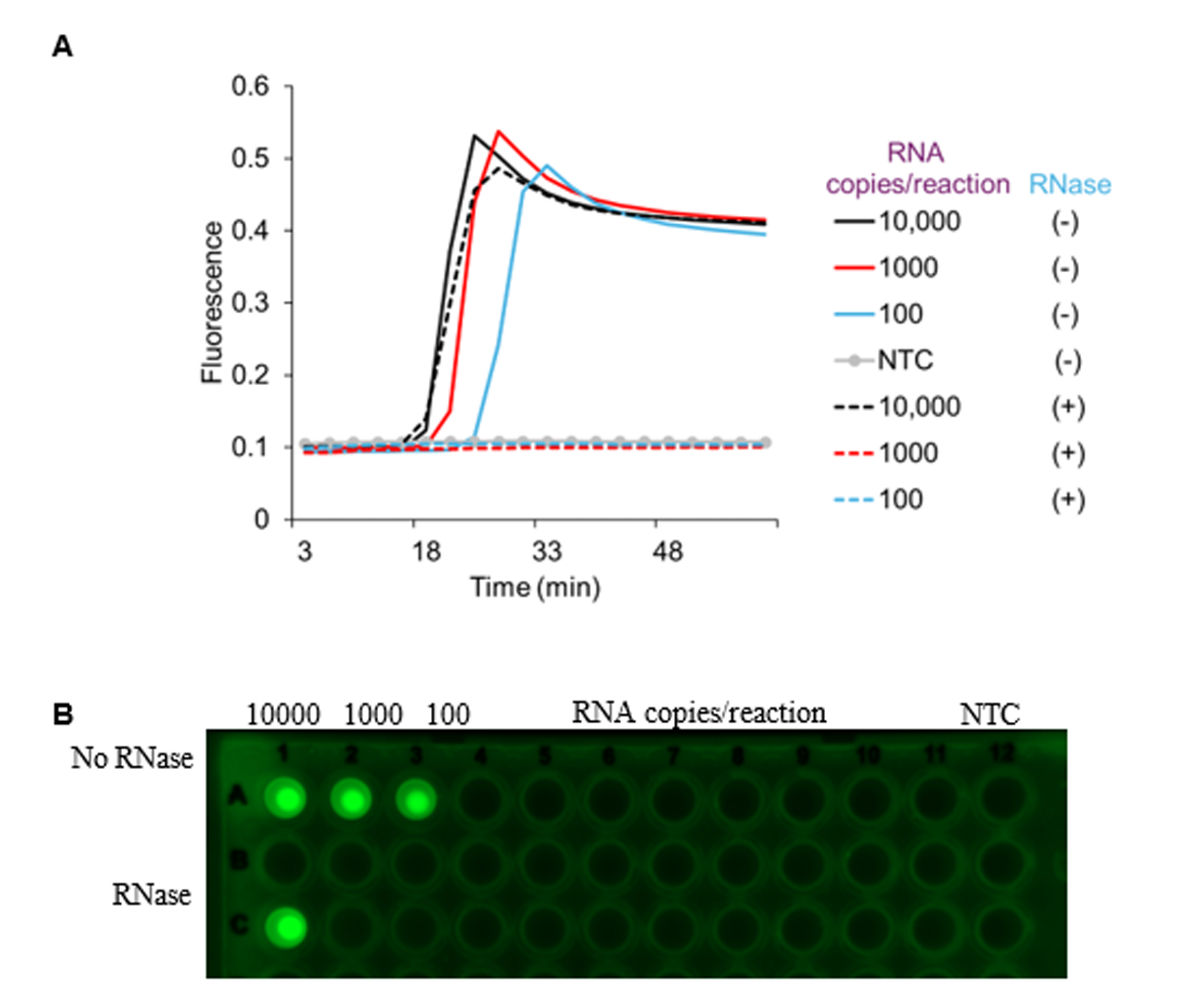

Supplement: Supplemental Information 10 — Indicated copies of 23S rRNA transcripts were analyzed using LAMP-OSD amplification before (solid lines) or after (dashed lines) treatment of the RNA templates with RNase cocktail for 1 h. Real-time measurement of OSD fluorescence accumulation in response to LAMP amplification of the Enterococcus RNA templates is depicted in panel A and endpoint imaging of OSD fluorescence in the same wells performed at LAMP reaction completion is depicted in panel B. [file peerj-14-21310-s010.png]

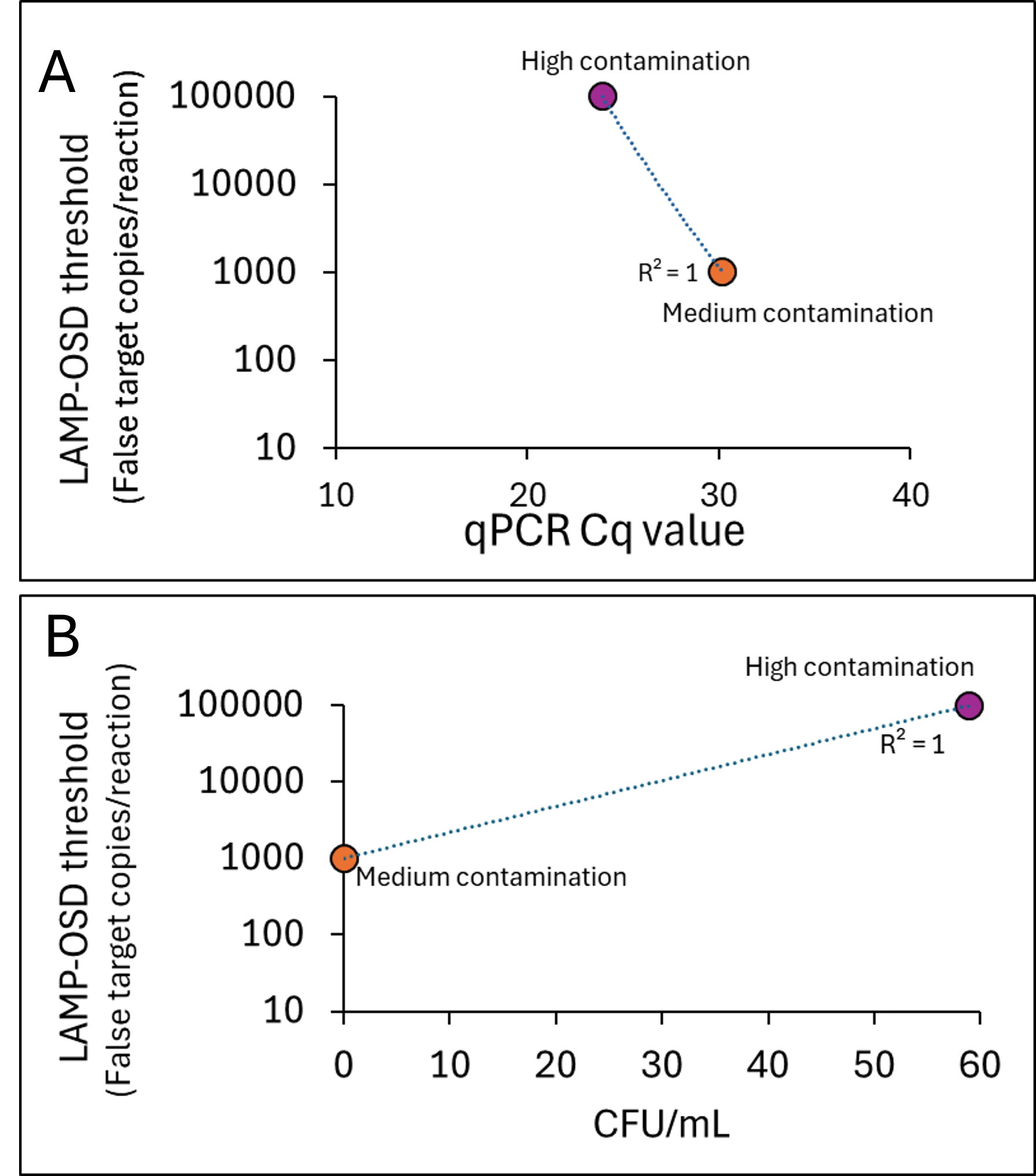

Supplement: Supplemental Information 11 — Scatter plots of data shown in Figure 4 depicting correlation between LAMP-OSD false target thresholds for visual detection of enterococci contamination level in environmental water and qPCR Cq values (A) or enterococci mEI plate counts (B). [file peerj-14-21310-s011.png]

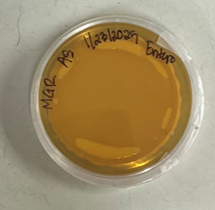

Supplement: Supplemental Information 19 [file peerj-14-21310-s019.png]

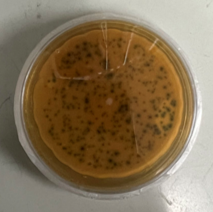

Supplement: Supplemental Information 20 [file peerj-14-21310-s020.png]

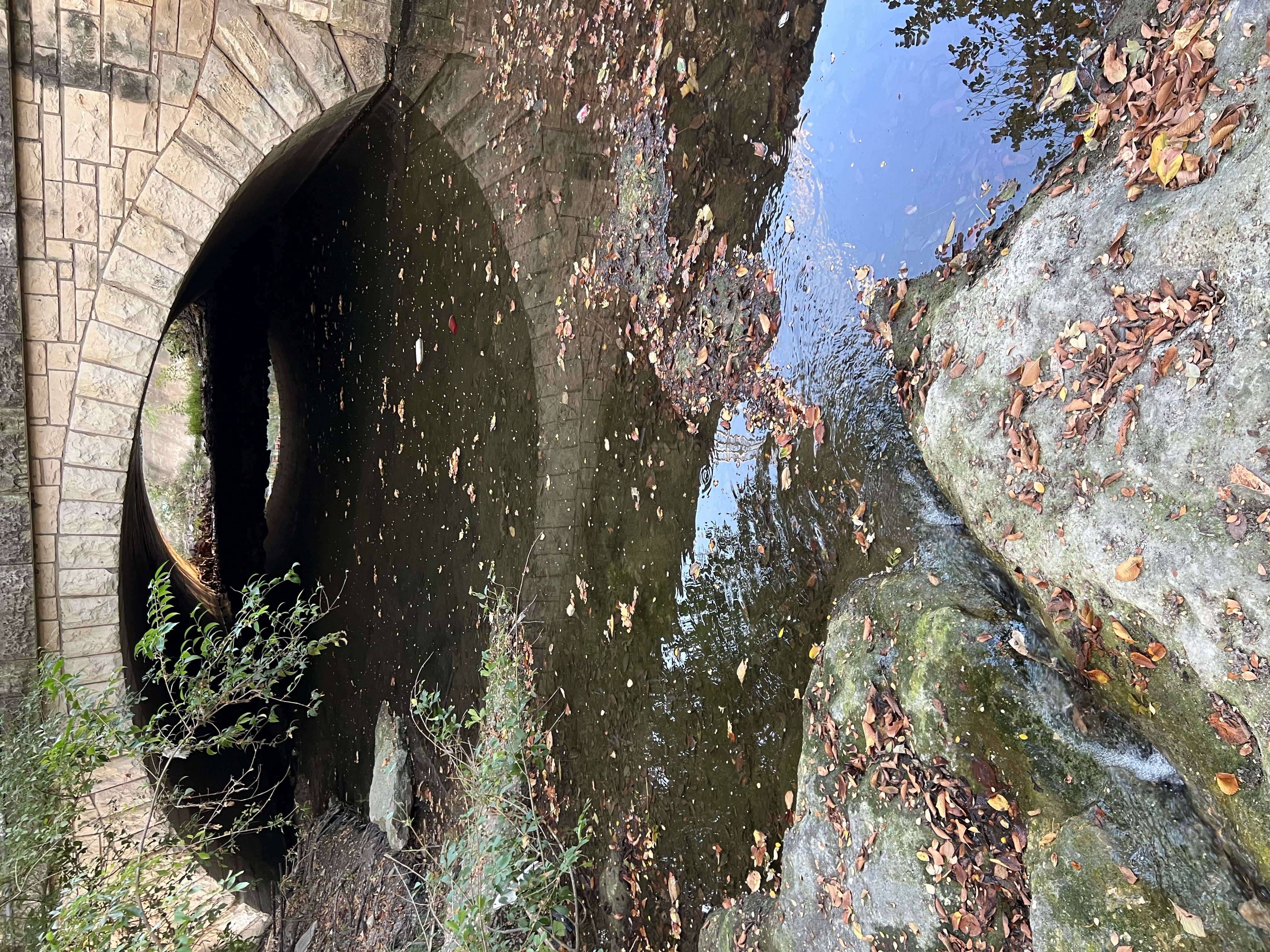

Supplement: Supplemental Information 21 [file peerj-14-21310-s021.jpeg]
